# The Global Flood Protection Benefits of Mangroves

**DOI:** 10.1038/s41598-020-61136-6

**Published:** 2020-03-10

**Authors:** Pelayo Menéndez, Iñigo J. Losada, Saul Torres-Ortega, Siddharth Narayan, Michael W. Beck

**Affiliations:** 1 IHCantabria - Instituto de Hidráulica Ambiental de la Universidad de Cantabria, 39011 Santander, Spain; 2 Institute of Marine Sciences, University California, Santa Cruz, CA 95062 USA; 3 The Nature Conservancy, Santa Cruz, CA 95062 USA; 4 Department of Coastal Studies, East Carolina University, 850-NC 345, Wanchese, NC 27959 USA

**Keywords:** Environmental impact, Natural hazards, Physical oceanography

## Abstract

Coastal flood risks are rising rapidly. We provide high resolution estimates of the economic value of mangroves forests for flood risk reduction every 20 km worldwide. We develop a probabilistic, process-based valuation of the effects of mangroves on averting damages to people and property. We couple spatially-explicit 2-D hydrodynamic analyses with economic models, and find that mangroves provide flood protection benefits exceeding $US 65 billion per year. If mangroves were lost, 15 million more people would be flooded annually across the world. Some of the nations that receive the greatest economic benefits include the USA, China, India and Mexico. Vietnam, India and Bangladesh receive the greatest benefits in terms of people protected. Many (>45) 20-km coastal stretches particularly those near cities receive more than $US 250 million annually in flood protection benefits from mangroves. These results demonstrate the value of mangroves as natural coastal defenses at global, national and local scales, which can inform incentives for mangrove conservation and restoration in development, climate adaptation, disaster risk reduction and insurance.

## Introduction

Coastal flooding impacts are increasing due to coastal development, population growth^1^, climate change^2,3^, and habitat loss^4–7^. In 2017 alone, overall storm damages were more than $US 170 billion in the North Atlantic^8^. However, development choices often neglect flood risks^3,9,10^ and there is growing pressure to adopt flood mitigation and adaptation strategies to reduce these impacts and economic losses^9,11,12^.

In many tropical and subtropical regions mangroves reduce waves and storm surges, and serve as a first line of defense against flooding and erosion. These benefits are provided through bottom friction, the cross-shore width of forests, tree density and shape. The aerial roots of a mangroves forest retain sediments, stabilizing the soil of intertidal areas and reducing erosion^13^. Roots, trunk and canopy dissipates storm surge^14^ and waves^15^. Previous studies have shown that mangroves can reduce up to 66% of wave energy in the first 100 m of forest width^15,16^. Mangroves can also provide adaptive defenses as they can, under the right conditions, keep pace with sea-level-rise through vertical accretion^17–19^.

Yet, mangroves have experienced significant losses over the last decades, declining globally from 139,777 km^2^ in 2000 to 131,931 km^2^ in 2014^20^, with even greater losses before 2000. Most of this loss has happened through the conversion for aquaculture or agriculture and coastal development^21^. The loss of these habitats can contribute to increasing coastal risk^22^, particularly in developed areas with great exposure of coastal populations^23,24^. Quantifying the value of mangroves as natural coastal defenses is crucial for incentivizing their conservation and restoration for the benefit of nature and people^25^.

The economic value of mangroves for services that rely on conserving them, such as flood protection, is typically not included within national budgets and wealth accounts^26^ in contrast to other services such as timber production. Estimates of flood protection benefits have been traditionally limited to local^27,28^ and national^29^ scale analysis. There are very few global estimates of ecosystem services from wetlands^30,31^, and none are based on process-based hydrodynamic flood models. Further, most assessments of the value of mangroves use a benefit transfer or replacement cost method^32,33^, instead of process-based methods that can account for local variation in characteristics of storms, mangrove habitat, topography and bathymetry. Field and numerical studies have shown that the capacity of mangroves to act as natural defenses vary considerably depending on environmental variables from the sources of flooding in the ocean to mangrove characteristics, coastal topography and also the inland receptors of damage^34^.

In a first, we provide a global analysis of the social and economic value of mangroves for flood risk reduction. This work is based on the approaches developed in previous research papers^29,34,35^ and the recommendations of the World Bank^36^, i.e.: (i) the use of process-based models; (ii) the application of the expected damage function approach for estimation of damages^37,38^; and (iii) the assessment of benefits by measuring the flood damages that mangroves avert^39,40^.

We assessed the total expected annual benefits of mangroves considering both cyclonic (“tropical cyclones”) and non-cyclonic (“regular”) conditions. Global mangrove benefits are quantified by estimating the difference in flood damages between two scenarios: (i) “with mangroves” (current global extent of mangroves) and (ii) “without mangroves”. For the two scenarios, we use rigorous process-based models to quantify the coastal flood extents and heights for various storm return periods. We assess the people and property damaged with and without mangroves across 700,000 km of mangrove coastlines globally. The difference between scenarios is the averted damages or benefits provided by current mangroves. We estimate the extent of inland flooding at 30-m resolution globally. For each mangrove scenario, these values are summarized in terms of expected annual damages, a metric that expresses the probability of expected dalmages in any year across the full spectrum of storms. The benefits of mangroves are assessed by the flood damages averted or avoided.

## Results

This probabilistic analysis identifies the places most sensitive in terms of coastal risk to the loss of mangroves at 30-m resolution, and aggregate results at global-scale, national-scale (countries) and local-scale (20-km coastal units).

### Mangroves and global flood reduction benefits

**Figure 1:**
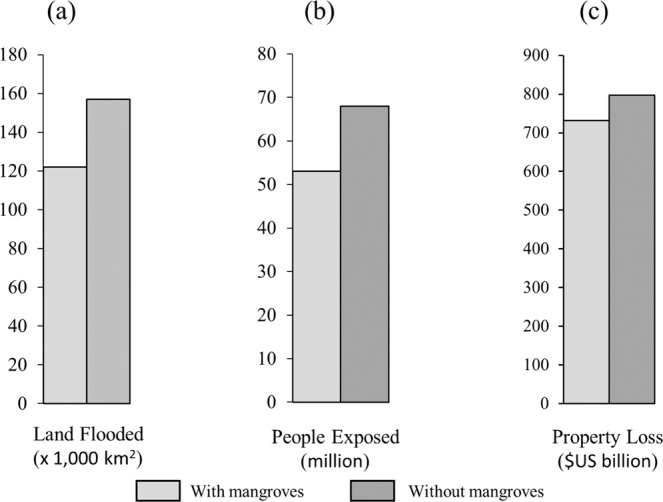
Annual expected benefits from mangroves for flood protection. Estimates of the effects of mangroves on avoided flooding to land (**a**), people (**b**) and property (**c**). The differences between scenarios with and without mangroves are the present flood protection benefits of the habitat.

**Figure 2:**
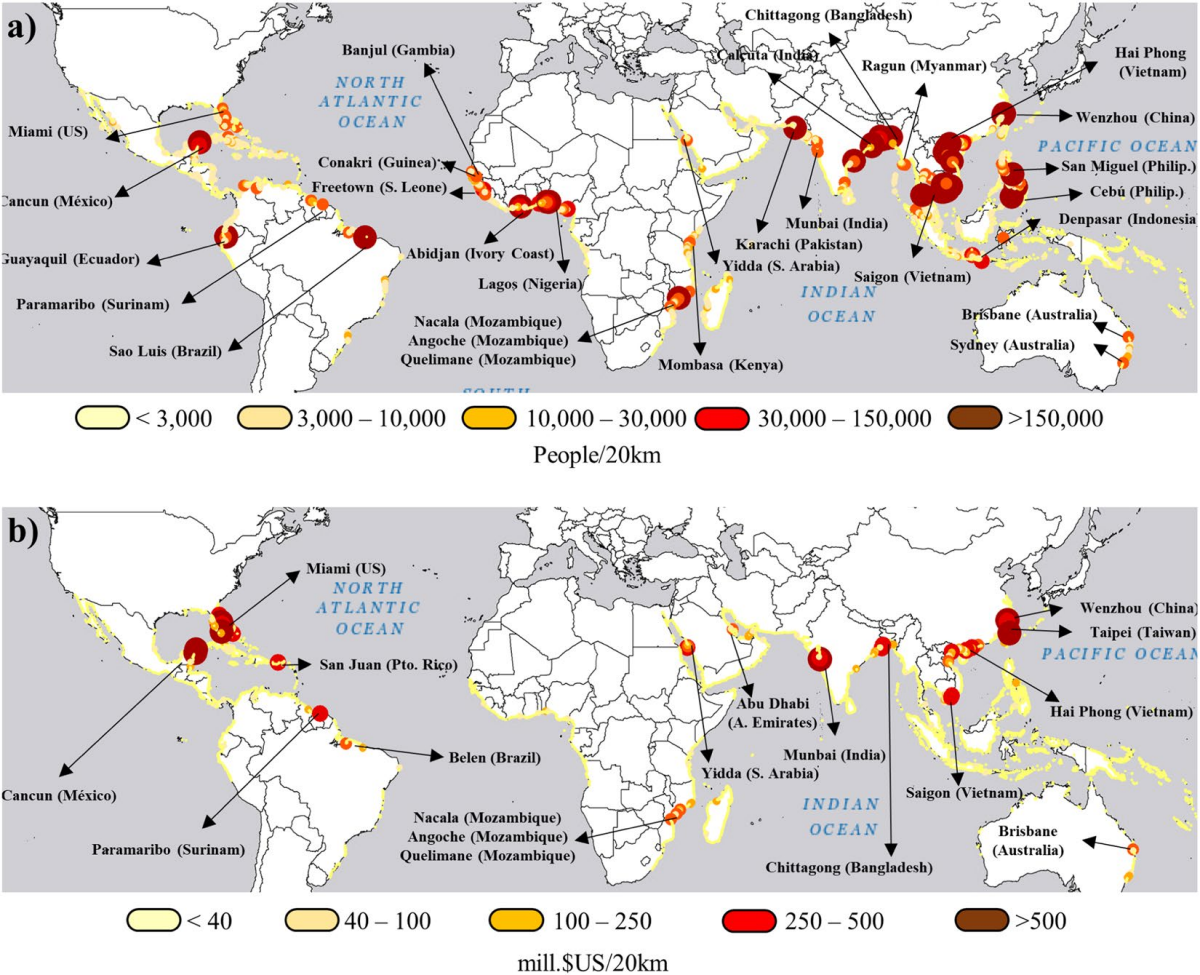
Annual expected benefits provided by mangroves to (a) people and (b) property per 20-km coastal unit. Base maps reprinted from ArcGIS Online maps under a CC BY license, with permission from Esri, original Copyright © 2018 Esri (Basemaps supported by Esri, DigitalGlobe, GeoEye, Earthstar Geographics, CNES/Airbus Ds, USDA, AEX, Getmapping, Aerogrid, IGN, IGP, swisstopo, and the GIS User Community).

Mangroves annually reduce property damage by more than $US 65 billion and protect more than 15 million people. If current mangroves were lost 29% more land, 28% more people and 9% more property would be damaged every year (Fig. 1). These values and benefits can be much higher locally (Fig. 2).

The percent risk reduction benefit provided by mangroves is relatively consistent across different return periods with a trend towards greater benefits for the more intense events (1-in-100-year), except for people protected. For example, property savings go from 7.8% (1-in-10-year) to 9.9% (1-in-100-year). Same patterns are observed in land flooded reduction (25.6–29.8%). However, the percentage of people protected from 1-in-10-year is greater than from 1-in-100-year (25.6% vs 19.3%) (Table 1).

**Table 1:** Global Benefits of Mangroves in Averted Flooding and Damages.

	Land flooded (x1000 km^2^)			People affected (million)			Property loss ($US billion)		
	With	Without	Benefit	With	Without	Benefit	With	Without	Benefit
Annual Expected	122	157	35	53	68	15	732	797	65
10-yr	176	221	45	82	103	21	1200	1293	93
25-yr	209	262	53	107	129	22	1558	1662	104
50-yr	249	318	69	138	166	28	1953	2092	139
100-yr	326	423	97	192	229	37	2714	2984	270

Values are the flooded land and people and damages to property with and without mangroves annually and for catastrophic events. The difference in flooding and damages is the benefit provided by mangroves. The catastrophic events are, for example, the storm event with a 1 in 10-yr return period (“10-yr”).

Approximately 90% of total benefits of mangroves are for protection from tropical cyclones, while 10% are from protection from regular (non-cyclonic) conditions (Supplementary Tables 1 and 2). For example, mangroves reduce annual expected flood damages from tropical cyclones by $US 60 billion and protect 14 million people (Supplementary Table 1). Meanwhile, they reduce global flooding from regular conditions by $US 5 billion and 1 million people every year (Supplementary Table 2).

In general, the benefits from mangroves increase as the return period increases, becoming more valuable during more intense events (i.e., 1-in-100-year) which are rare but cause significant flood damages (Table 1). If mangroves were lost, property losses produced by 1-in-100-year flood events would increase by 37 million people and US$ 270 billion (Table 1). However, for tropical cyclones, mangrove benefits increase sharply after reaching a storm intensity associated to the 1-in-50-year return period events (Supplementary Table 1).

### Mangroves and national flood reduction benefits

The flood protection benefits of mangroves vary significantly across regions and countries due to differences in flood characteristics, mangroves extents and the degree of exposure. Overall mangroves provide the greatest benefits in the Western Pacific and Caribbean islands world (Table 2 and Supplementary Figs. 1–3). The countries that receive the greatest annual economic benefits from mangroves are typically more developed states and territories: United States, China and Taiwan. These areas principally benefit from mangroves in terms of the high value and density of coastal assets that are protected. Vietnam, India and Bangladesh benefit the most from mangroves in terms of people protected due to the high density of coastal populations in these countries (Table 2).

**Table 2:** Country ranking.

(a) Land [x1,000 km^2^]			(b) People [million]			(c) Property [$US billion]			(d) Property/GDP [%]		
1	Cuba	3.92	1	Vietnam	7.02	1	United States	11.31	1	Belize	28.86
2	Vietnam	3.12	2	India	2.87	2	China	8.58	2	Suriname	21.35
3	Bahamas	2.47	3	Bangladesh	1.11	3	Taiwan	7.89	3	Mozambique	17.59
4	Cambodia	1.78	4	Philippines	0.61	4	India	7.84	4	Bahamas	13.72
5	India	1.63	5	China	0.52	5	Mexico	7.42	5	Anguilla	4.63
6	United States	1.42	6	Brazil	0.33	6	Vietnam	6.45	6	Guyana	4.57
7	Nicaragua	1.40	7	Nigeria	0.30	7	Mozambique	1.94	7	Madagascar	3.57
8	Mexico	1.13	8	Indonesia	0.25	8	Saudi Arabia	1.61	8	Guinea Bissau	3.24
9	Honduras	1.07	9	Mozambique	0.24	9	Bangladesh	1.56	9	Vietnam	3.14
10	Indonesia	0.84	10	Mexico	0.23	10	Bahamas	1.55	10	Turks and Caicos	2.57
11	Bangladesh	0.82	11	Ivory Coast	0.21	11	Philippines	1.00	11	Sierra Leone	2.02
12	Brazil	0.76	12	Thailand	0.18	12	Australia	0.79	12	Taiwan	1.71
13	Guyana	0.75	13	Ecuador	0.18	13	UAE	0.74	13	New Caledonia	1.16
14	Belize	0.71	14	Taiwan	0.17	14	Brazil	0.72	14	Solomon Islands	1.07
15	Madagascar	0.69	15	Pakistan	0.14	15	Suriname	0.70	15	Ant. & Barbuda	1.06

The countries receiving the greatest benefits from mangroves in averted land flooding and damages to people and property. The table also shows the benefits of mangroves (averted flood damages to property) relative to GDP.

Indeed, the national importance of mangroves for flood protection varies considerably when considering these benefits as a percentage of national GDP. For example, in Belize, Suriname and Mozambique, the flood protection benefits from mangroves account for over 15% of the national GDP. Mangroves provide critical flood protection benefits in countries with lower GDPs where exposure is concentrated along vulnerable coastlines; for example, Mozambique and Bangladesh, 7th and 9th respectively in terms of mangroves benefits. These countries receive over $US 1 billion in benefits annually from mangroves due to the high densities of assets in exposed coastal areas (see Supplementary Fig. 3 for relative property benefits distribution).

The benefits of mangroves from cyclones are particularly high for countries such as Mexico, India and Vietnam. For countries, where cyclones are not as common such Japan and China, mangroves can still provide significant benefits from more common high waves and swell. There are also nations (e.g. Australia and United Arab Emirates) where mangroves protect the same from tropical cyclones and regular climate (Supplementary Tables 3 and 4).

### Mangroves and local flood reduction benefits

Mangroves also provide significant flood protection benefits to several coastal cities and regions (Fig. 2). In many of these cities, mangroves protect a considerable number of people from flooding annually (Fig. 2a). For example, in Abidjan and Lagos in West Africa, Mumbai and Karachi in South Asia, Wenzhou in East Asia, and Cebu and Denpasar in South-east Asia existing mangroves protect more than 150,000 people from flooding every year. In some cities like Miami in the U.S.A and Cancun in Mexico mangroves provide more than $US 500 million in avoided property damages every year (Fig. 2b). However, mangrove benefits are not limited to urban areas and extend to less populated coastal floodplains.

## Discussion

This study provides the first global analysis of the economic value of mangroves for flood protection. Where they remain, mangroves reduce risks by protecting coastlines against flooding from waves and storm surge. They protect lives, prevent damages to assets critical to livelihoods and reduce socio-economic vulnerability. Many important benefits do go to developed nations particularly to some of those with smaller economies (lower GDP) that are least able to respond to disasters. Mangroves forests around the world have faced extensive loss and degradation due to ditching, loss to open water or conversion to other land-uses^20^. By quantifying the value of mangroves in terms of economic benefits to people and property globally, this study helps demonstrate the importance of conserving mangroves where they exist today. While global scale results are best suited for identifying hotspots in services provided by country or region^34^, local and national levels are appropriate for project design, implementation and cost-benefit analysis^29^.

Mangroves provide significant annual flood protection savings for people and property both from cyclones and the more regular (non-cyclonic) high wave and swell events. However, cyclonic events are when damages are the greatest and mangroves offer the greatest benefit. With climate change the intensity and frequency of the largest events are likely to increase and thus the role of mangroves will therefore be even more relevant in future scenarios.

The greatest economic benefits are received by USA and China. These are highly developed nations where mangroves have been severely degraded by coastal development. Nonetheless the remaining mangroves provide significant values annually in states, territories and provinces such as Florida (USA). Developing countries and small islands are the most vulnerable to mangroves loss. These countries receive benefit from the greatest economic protection relative to the GDP (e.g. Belize, Suriname, Mozambique, Bahamas, Anguilla, Guyana and Madagascar). The influence of mangroves on flooding varies spatially at a national level. Mangroves in some countries have an apparently greater effect on flooding due to unique combinations of hazard, ecosystem and exposure characteristics. For example, while the total extent of mangroves in Indonesia is nearly 6.5 times that of Cuba^20^, Cuba receives significantly higher protection from mangroves in terms of flood extents (4.5 times more land protected). This discrepancy can be explained by the fact that the value of mangroves for flood protection depends significantly on the coastal length of mangroves even more than the width of the mangroves forest. Previous studies have shown that the flood reduction benefits from mangroves and other coastal wetlands, particularly from waves, are highly non-linear on forest width^35,41^. This implies that coastlines with longer mangroves belts, such as in Cuba, may benefit more in terms of flood reduction^42^. Differences in these results can also arise due to differences in inland topography behind these mangroves. Mangrove benefits tend to be higher on flatter floodplains where storm surges travel far, relative to steeper floodplains.

By showing where mangroves are most valuable in terms of both people and property protected, this study provides important insights for where to prioritize restoration efforts. Local scale analysis highlights the hotspots where mangroves provide the greatest benefits. For example, mangroves provide relevant benefits throughout the Philippines, but these values are higher in the central and northern regions of the country, as they are the areas that receive the greatest annual impact from typhoons. In addition, mangroves provide benefits especially in densely populated lowland areas, such as in the Ganges-Brahmaputra delta in India and Bangladesh; also, in the Mekong delta in Vietnam; or in the Amazon delta in northern Brazil. These regions are highly sensitive to climate hazards and therefore need specific risk reduction strategies (e.g. UNISDR 2015^11^). It is in the most vulnerable areas where mangroves play the most important role in reducing risk by minimizing flood exposure and, therefore, the number of people likely to be affected by such events. Mangroves were and are often filled, ditched, diked and dredged to build coastal infrastructure from airports to ports, hotels and housing developments. In these areas few mangroves remain in front of these properties to provide protections. Remaining mangroves however particularly protect communities and sometimes the most socially vulnerable communities at least with respect to poverty and income^29^.

Our flood maps “with mangroves” provide some of the current best risk assessments available for many countries (e.g. Supplementary Figs. 4 and 5). This global flood risk analysis improves on earlier global flooding analyses^3,9,10^. Our work is based on a fully probabilistic approach and we followed a multistep methodology based on process models, in combination with statistical downscaling, to simulate wave and surge interaction with mangroves and predict flood impacts along the coast. We examine waves and surge in both cyclonic and more regular (non- cyclonic) conditions. We assess flooding of land at very high resolution (flood maps and risk maps at 30-m resolution worldwide). Valuing mangroves at global, national and local scales provides a consistent screening of the magnitude of ecosystem benefits, allows to identify the greatest nature-dependent areas (priority management zones) and highlights the cost-efficient solutions.

To assess flooding globally, we make a number of key assumptions and simplifications, which are summarized here and cover in depth in Supplementary Table 9. We developed and validated a key storm model with high-resolution analyses for the Philippines^29,43^. A global reanalysis of tropical cyclone storm surges has not been available during the development of this work, we developed and validated a regression model based on the country with the broadest range of storm intensities, mangroves characteristics and coastline (Supplementary Fig. 7). Other regional studies that include tropical cyclone reanalysis underestimate storm surge (e.g. Haiyan typhoon)^44^. However, our model is able to accurately capture these extreme events, as we demonstrated in the Philippines (Supplementary Fig. 11).

In the future, all coastal flood risk models will be improved by better data on bathymetry, topography, and mangroves as well as better models of the two-dimensional propagations of nearshore waves and storm surge. For consistency and computational savings, we have used global datasets and time-efficient modeling tools. We have examined issues of model sensitivities in depth elsewhere^43^. Because this is a global flooding model, we excluded some countries that had very few mangroves (less than 100 ha) and we also capped the benefits per hectare at $US 50,000 as these were the highest values estimated in a high resolution analysis, risk industry model of mangrove benefits in Florida (Narayan *et al*. 2019) (see Supplementary Table 8). This excluded 15 countries in total, including Bahrain and Benin, which had very high values of benefits/ha; as well as eight Caribbean Small Island Developing States (Supplementary Table 9).

These models and results inform new opportunities to pay for the management, conservation and restoration of mangroves to cost effectively reduce risks to people and property. There is strong interest among the management, financing and donor sectors for solutions in disaster risk reduction and climate adaptation particularly as payments from national governments and insurers are growing nearly exponentially for disaster management^45^. Many governments subsidize risk, which creates perverse incentives for greater coastal development, loss of ecosystems, and reduced opportunities for private insurance. By quantifying the values of coastal mangroves, this opens opportunities to align their conservation with coastal protection of existing public infrastructure and private developments.

The approaches we use here for assessing flood risk and the benefits of mangroves as risk reduction solutions are consistent with those used by national disaster risk agencies (e.g., the US Federal Emergency Management Agency), coastal engineers (e.g. the U.S. Army Corps of Engineers), private insurers and re-insurers, climate adaption funders (e.g., Green Climate Fund), and the World Bank^46,47^. By using widely accepted approaches to measure the benefits and cost-effectiveness of mangroves, these results open opportunities to support the management and restoration of mangroves as national coastal infrastructure using hazard mitigation and disaster recovery funds. These values can also be used to underpin the development of innovative insurance options like those being developed and implemented for coral reefs^45,48,49^. These spatially explicit values can be used directly in national adaptation and risk management plans associated with the United Nations Conventions on Climate Change and Disaster Risk Reduction (UNFCCC and UNSIDR). These values could also be used to inform the development of resilience credits for climate adaptation, similar to the development of credits for blue carbon (for climate mitigation) in mangroves. By demonstrating where mangroves provide flood protection benefits across the world, this study helps inform wider discussions on where it is most optimal to invest in efforts to restore and manage mangroves for the critical ecosystem services they provide.

## Methods

### Methods at a glance

This work measures the flood protection service of mangroves all over the world for two climatic conditions: (1) Cyclonic- i.e., the conditions high-intensity extreme waves and storm surge induced by tropical cyclones and (2) Non-cyclonic, i.e., the “regular” waves generated by low-intensity local storms. We followed the Expected Damage Function (EDF) approach^50^, recommended by the World Bank^36^, previously applied in coral reefs ecosystems^34^ and commonly used in engineering and insurance sectors^51,52^. We examine the role of mangroves in reducing flood risks by measuring the impacts of flooding on people and property under two different scenarios: with and without mangroves. The “without mangroves” scenario assumes the complete loss of mangrove habitat and the consequent erosion of the intertidal area with a smoothened bottom roughness. We use a regression model globally, to calculate coastal flooding by analyzing more than 7,000 historical cyclones^53^ and 32 years of regular waves and sea level (storm surge, astronomical tide and mean sea level). Flood impacts (i.e. land flooded) for the with and without mangrove scenarios are combined with global distributions of people and property^11^, and with vulnerability based on global “Flood Depth-Damage Functions”^54^ to assess baseline flood damages and flood damages after mangrove loss for multiple storm events and on an annual basis.

To identify the mangroves that influence a given coastal region and evaluating nearshore hydrodynamics and flood height we define cross-shore coastal profiles (Supplementary Fig. 6d). Then, we follow a multi-step framework whose key aspects are described here and in the Supplementary Material: (1) Estimate offshore dynamics produced from both, tropical cyclones and regular climate conditions. (2) Estimate nearshore dynamics by downscaling offshore waves and storm surge until shallow water, just before mangrove habitats. (3) Propagate waves and storm surge through mangroves and obtain the flood height behind the mangroves at the shoreward end of each profile. (4) Estimate the land flooded (impact) due to extreme water levels along the shore by intersecting the flood height at the shoreline with inland topography (5) Calculate land, people and property located in the flooded area and, finally, apply the corresponding damage functions to obtain flood damages with and without mangroves.

### Study domain description

This global study covers 700,000 km of coastline that includes more than 141,000 km^2^ of mangroves, spread over 4 continents and more than 9,500 islands. To reduce the vast computational requirements such a large domain requires, the global domain is divided at three levels, from global to regional and local (Supplementary Fig. 6). The first level is a global division into six macro-regions corresponding to the following ocean basins of tropical cyclone generation^53^: East Pacific, North Atlantic, North Indian, South Indian, West Pacific and South Pacific (Supplementary Fig. 6, panel “a”). The second level divides the 700,000 km of global mangrove coastline into 68 sub-regions considering coastline transects of similar coastal typology (e.g. islands or continental coasts) and similar ecosystem characteristics (Supplementary Fig. 6, panel “b”). The third level of disaggregation is done at a local scale, defining units with 20-km length of coastline and extending up to 30-km inland and 10 km seaward (Supplementary Fig. 6, panel “c”). Within these units cross-shore profiles perpendicular to the mangrove habitats are created for each kilometer of mangrove coastline, totaling 700,000 profiles (Supplementary Fig. 6, panel “d”).

### Building the global model based on the Philippines results

Global reanalysis of ocean and coastal waves^55,56^ and storm surge^57^ exist though not for tropical cyclones during the course of this work. We develop the global model based on an extensive re-analysis of tropical cyclone climates developed for the Philippines^29^. We chose the Philippines as the baseline case to develop our own tropical cyclone reanalysis at high resolution and estimate flood damages in presence and absence of mangroves. There are three main reasons that make the Philippines an excellent pilot case for valuation of the coastal protection ecosystem service provided by mangroves: (i) Almost 10% (548 events) of the global tropical cyclone records from IBTrACS database affected the Philippines^53^. The worldwide distribution of tropical cyclone parameters (velocity track, wind speed) closely resemble these events in the Philippines (Supplementary Fig. 7) (ii) The islands of the Philippines present high climatic variability and it is at particularly risk from natural hazards like typhoons and regular storms, which are the cause of 80% of the total losses from disasters [average loss totaling nearly $US 3 billion, 29% of this damage is due to coastal flooding^58^]. (iii) The Philippines ranks in the top 15 most mangrove habitat-rich countries, with 2,630 $$k{m}^{2}$$ in 2010, representing 2% of the world total^59^. These mangrove habitats show extensive variation in both cross-shore length and average depth. Mangroves in the Philippines rage between 0.1 km and 8 km wide and between 0 m and 10 m depth (Supplementary Figs. 8 and 9). We valued flood protection service of mangroves in the Philippines by using the numerical model Delft3D considering both historical tropical cyclones and regular climate conditions with and without mangroves. We use these results to build two global statistical models. The first global model is developed to obtain offshore and nearshore ocean dynamics produced by tropical cyclones (wave height, peak period, storm surge and storm duration), and the second global model to estimate how the presence and profile of mangrove habitats influence the total water level on the shoreline. Further details of the two models are developed below:

#### Model 1: Offshore and nearshore dynamics generated by tropical cyclones

Offshore waves and storm surge generated by tropical cyclones (IBTrACS database) were numerically simulated in the Philippines by using Delft3D modules “Flow”^60^ and “Wave”^61^. Both modules were run simultaneously in a 2-dimensional grid of 5 km cell-size with a time step of 30 s, forced with hourly wind data and sea level pressure fields obtained from parametric model, in which the non-linear interaction processes of tide, wind setup, inverse barometers and wave setup are considered. The model was validated by comparing the storm surge generated by typhoon Rammasun, in Legaspi and Subic Bay. We use tidal gauges registers from the Global Sea Level Observing System (GLOSS, http://www.gloss-sealevel.org) for validation (Supplementary Fig. 11). Using the results of the numerical simulations carried out with the Delft3D model in the Philippines we look for statistical relationships between cyclone parameters and oceanographic variables to create a new predictive model, where oceanographic variables (wave height, period, weather tide and duration of the storm peak) are predicted based on cyclone parameters (distance, wind speed, track velocity, wind angle of incidence). In the Philippines, 548 events were simulated on a two-dimensional grid of 5 km cell size, finally creating a database of 58 million results. We randomly select 90% of the generated results to build our predictive model, and use the other 10% for validating the predictive models. We estimate the correlation between physical tropical cyclone parameters (distance from the trace to the profile *D* [km], wind speed *W* [km/h], cyclone travel speed *V* [km/h], wind direction from north *θ*_*WN*_, [in degrees] and the angle between the wind direction-profile *θ*_*WP*_ [in degrees]) and the oceanographic variables at the target point (maximum significant wave height produced during the event at the target point *H*_*smax*_ [in m], peak period *Tp* [in s], maximum storm surge *SS*_*max*_ [in m] and maximum storm surge duration, *T*_*SSmax*_). We increase the accuracy of the analysis by dividing the data into two groups: Coastal areas directly exposed to tropical cyclones and areas protected from the direct impact of tropical cyclones (Supplementary Fig. 13). For each combination (5 cyclone variables x 3-time instants x 4 oceanographic variables = 60 cases), we estimate the Pearson coefficient (P_xy_), which statistically quantifies the degree of correlation between the cyclone variables “X” and the oceanographic variables “Y” (equation S.1). We then adjust ocean climate variables (Yi) to our parametric model [equation S.2]. We test this adjustment for one, two, three and four independent variables (Xi), so that we can cover all the alternatives and, based on the correlation coefficient of each one, choose the best regression model.

1 $${{\rm{Y}}}_{{\rm{s}}}={{\rm{a}}}_{0}+{{\rm{a}}}_{1}\cdot {{{\rm{X}}}_{1}}^{{\rm{\alpha }}1}+{{\rm{a}}}_{2}\cdot {{{\rm{X}}}_{2}}^{{\rm{\alpha }}2}+\ldots +{{\rm{a}}}_{{\rm{n}}}\cdot {{{\rm{X}}}_{{\rm{n}}}}^{{\rm{\alpha }}{\rm{n}}}$$

Where Y could be either the maximum wave height (Hs_max_), the peak period (Tp), the maximum meteorological tide (SS_max_) and the duration of the meteorological peak produced by the cyclone (Tss_max_). Meanwhile, X could be any of the following predictor variables (see equations S.3 to S.10): minimum distance between the storm track and the target point (D_min_), the wind speed when the tropical cyclone is at the closest location to the target point (W_dist_min_), the average wind speed during along the storm length (W_mean_), the mean direction of wind respect to the North (θ_WN_mean_), the wind direction respect to the North at the minimum distance point (θ_WN_dist_min_), the average track velocity (V_mean_), the track velocity at the minimum distance point (V_dist_min_) and is the track velocity at the moment of maximum wind speed (V_wind_max_).

#### Model 2: The role of coastal habitats in nearshore dynamics (flood height)

Coastal vegetation provides resistance to the energy and flow of waves and water as they come onshore which is modeled by using a friction factor. Mangroves are then modeled in terms of an equivalent roughness [e.g. Sheppard friction for coral reefs^62^] based on Manning coefficient. In the Philippines we classify surface types into three groups: sandy soil (n = 0.02)^63^, mangroves (n = 0.14)^63^ and coral reefs (n = 0.05)^64^. 1-dimensional numerical propagations are carried out using the Delft3D model to obtain flood heights along the coast (Supplementary Figs. 8 and 9). We use these numerical results to create two interpolation tables (for both, regular climate and tropical cyclones) that correlate the climatic information at seaward side of the profile (Hs, Tp, SS and Tss, being this last only specific of tropical cyclones) and the characteristics of the mangrove profiles (width and average depth) with the flood height (i.e. total water level along the coast). These tables contain 37,500 tropical cyclone simulations (50 cyclones × 750 profiles) and 90,000 regular climate simulations (120 sea states × 750 profiles).

These two models described above are integrated in the multi-step framework applied globally for valuing the flood protection role of mangroves:

### Step 1: Offshore dynamics

The offshore hydrodynamic conditions (wave height, wave period, storm surge and astronomical tide) were subdivided in two groups: (1) those produced by local extreme events (tropical cyclones) and (2) those produced by less intense local climate or extreme climate generated far away from the study area (regular climate). **Regular climate** is defined by different datasets within the period 1979–2010: a global wave reanalysis^56^, a global storm surge reanalysis^57^, astronomical tide^65,66^ and mean sea level compiled from historical numerical reconstruction and satellite altimetry^67^. Waves and sea level conditions due to tropical cyclones are excluded and studied separately to avoid double counting. **Tropical cyclones** were considered separately from regular climate only if two conditions are satisfied: (1) they are generated within the same ocean basin than the study area and the cyclone passes closer than 500 km from the coastline, and (2) 10-minute sustained wind speeds (W_10m_) exceed 118 km/h. Tropical depressions (W_10m_ ≤ 62 km/h) and tropical storms (63 km/h ≤ W_10m_ ≤ 118 km/h) are studied together with regular climate. For historical tropical cyclones, we used IBTrACS database^53^, which provides 6-hourly data of wind speed, atmospheric pressure and position. Since global reanalysis of tropical cyclones that include waves do not exist, we use a statistical model (Equations S.3 to S.10) created from the Philippines results to calculate offshore wave height, peak period, storm surge and storm surge duration just in the limit between deep and shallow water.

### Step 2: Nearshore dynamics

Once we resolve offshore dynamics, we obtain waves and storm surge in the seaward side of each cross-shore profile. Waves interact with the bottom and other obstacles (e.g. islands) as they approach the coast and modify height and direction through shoaling, refraction, diffraction and breaking processes. **Regular climate** is propagated following a hybrid downscaling. The 32-year long series, from 1979 to 2010, include 280,000 sea states (1 sea state is 1-hour register of wave height, peak period and total water level). To each profile, we allocate the closest point of the offshore databases. Considering all, the 700,000 coastal profiles and the 280,000 sea states results in an unmanageable number of cases. We reduce the number of sea-state propagations by, firstly, considering only the 3,787 non-repeated combinations of wave height, peak period and total water level (SS + AT + MSL) and, then, applying The Maximum Dissimilarity Algorithm (MDA^68,69^) to finally obtain 120 sea states to be propagated with Snell law and shoaling equation (Eqs. 1 and 2). Meanwhile, **tropical cyclone** nearshore hydrodynamics are obtained by means of the previously derived regression model (equations S.3 to S.10). We apply regression models in each profile, and we obtain the same parameters as for regular climate, in addition to the time duration of the meteorological tide (T_ss_).

2 $${H}_{s\_perfil}={H}_{s\_off}\cdot \sqrt{\frac{{C}_{off}}{{C}_{perfil}}}\cdot \sqrt{\frac{\cos \,{\theta }_{off}}{\cos \,{\theta }_{perfil}}}$$

3 $${\rm{Snell}}:\frac{{C}_{off}}{\cos \,{\theta }_{off}}=\frac{{C}_{perfil}}{\cos \,{\theta }_{perfil}}$$

### Step 3: Modeling the role of coastal habitats in nearshore dynamics, flood height

The next step consists on propagating ocean hydrodynamics over mangroves forest which dissipate wave and surge energy, and, consequently, reduce flood height. Flood height is a function of mean sea level, astronomical tide and run-up of waves. Mangroves dissipation takes place by means of breaking and friction processes. Given the large scale of this global analysis, we follow a simplified approach for vegetation modeling. We use the interpolation table from the Philippines to infer the resulting flood height given mangrove length and depth, significant wave height, peak period and total water level at the head of each cross-shore profile. Then, we apply the statistical reconstruction technique RBF (Radial Basis Functions)^70^ to calculate in each profile the complete historical flood height time series. Next, we carry out an extreme value analysis. First, we select maximum values on a variable threshold (minimum, 1-in-5-year event). We adjust these selected values to a Generalized Pareto-Poisson distribution, and we obtain the flood height vs return period curves for both scenarios: with and without mangroves. We observe a high spatial variability of flood height produced by tropical cyclones along worldwide coastlines, which highlights the importance of addressing global flood risk analysis at high resolutions to consider local topographic and bathymetric variations (e.g. 1-in-100-year flood in Vietnam, Supplementary Fig. 10). We assume that countries with less than 100 ha of mangroves were excluded from the analyses as there were too few mangroves to reliably estimate benefits using a global model. This excluded 15 countries in total, including Bahrain and Benin, which had some of the most over-estimated values of benefit/ha; as well as eight Caribbean Small Island Developing States (Supplementary Table 9).

### Step 4: Calculating impacts: flooding maps

Uncoupling wave and surge propagation from the flooding process allows us to freely choose the most accurate flooding method. Other alternative strategies exist, but they are unapproachable at global scale due to its computational cost and high-quality data required, usually unavailable at global scale (e.g. using coupled phase resolving models or phase averaged models). To obtain flood maps by means of uncoupling propagation and flooding processes require: (i) the flood height along the coast with high enough resolution to avoid significant longshore gradients, (ii) a good DTM (Digital Terrain Model) and (iii) a flooding method (flood models). Separating flooding process from waves and sea level propagation gives us more flexibility to adapt the flooding approach to the elevation data. Local scale analyses (<100 km of coastline) with high resolution DTM (<10 m) could be addressed by using process-based flood models, like RFSM-EDA (Rapid Flood Spreading Method – Explicit Diffusion wave with Acceleration term)^71,72^. However, larger scale´s (>100 km) with coarser DTM (>10 m), require fast and less precise techniques, such as “bathtub” method, based on hydraulic connectivity which consists of merge points below the flood height. We use this last strategy to address global flooding in presence and absence of mangroves. The flood extent is estimated globally by using a 30-meter SRTM-DTM (Shuttle Radar Topography Mission)^73^.

### Step 5: Assessing global flood consequences in mangroves protected areas

Mangrove benefits are assessed in terms of avoided damages to people and property. Property value is directly obtained as the sum of industrial and residential stock from GAR15, at 5 km resolution worldwide^11^. The suitability of this database to be used in global assessments of coastal flooding exposure and damage lies on the fact that it integrates homogeneous global population and country‐specific building typology, use and value data^74^. The consistency of the methodological approach used in the development of GAR15, as well as the choice of the best data currently available for its implementation, have produced a product fully adapted to the needs of the global model of the evaluation of probabilistic risk^74^. Consequently, GAR15 is the most appropriate source of data available for global scale analysis, looking for an order of magnitude of the value of adequate protection for mangroves, usually used by critical stakeholders such as the World Bank^75^. People distribution comes from the freely available 1 km resolution database GPW (Gridded Population of the World), from SEDAC (Socioeconomic Data & Applications Center). To be consistent with flood layer grid resolution (30-m) it is necessary to redistribute people and property over a finer mesh. We apply a downscaling method, in which the re-distributed values are calibrated with the other existing data of people distribution (WorldPop) by imposing as boundary condition that the total sum of the assets re-scaled to 30 meters in each region was equal to the sum of those same assets without re-scaling in the same control zone. The sensitivity of people and stock to different levels of flooding is obtained through different damage functions. Damage functions provide information of the number of people affected by coastal flooding and the stock losses, according to the water depth. We use different damage functions for population and for stock. Population damage is based on the hypothesis that water depths below 0.5 meters do not affect people, while water depths above 0.5 meters affect 100% of people hit by flooding. It is a common practice in the scientific literature not to use damage functions to calculate the population affected by floods^3^. This option overestimates the results obtained; therefore, it is recommended to opt for a certain threshold below which the effects of flooding are not considered^54^. This threshold is set at 0.5 meters because it is a common value used by emergency services (Japan, Netherlands, USA) in determining whether or not it is necessary to evacuate people from an area under threat. In case of stock, we adapted the global flood depth-damage functions from Huizinga/JRC (Joint Research Centre) broken down by continent (Africa, Asia, Oceania, North America, South America and Central America) and by asset type: residential and industrial^54^. Average values of damage at different water depths are provided in Supplementary Table 5. Finally, flood risk (magnitude and probability) is obtained by combining damage curves with people and property exposure distribution. Then, we integrate the return period curves to obtain the Expected Annual Damages and Benefits at each 20-km study unit. We can thus show global information on annual flood damage anywhere and with a spatial resolution high enough to be incorporated into coastal planning and ecosystem conservation policies.

### Modeling assumptions

To provide a more nuance discussion of the strengths and weaknesses of the modelling approach, we have included a table with all the assumptions stablished at each step of the methodology, as well as the corresponding reference to the existing literature where this assumption is applied and validated (Supplementary Table 9). This table summarizes the assumptions considered in this work and may help the reader to assess how strong the assumptions are and potentially identify areas for future work.

## Supplementary information

Supplementary information.

## Data Availability

The data that support the findings of this study are available online at: https://osf.io/ecs4p/ (DOI 10.17605/OSF.IO/ECS4P).

## Code availability

The MATLAB codes for key analyses are available on request from the corresponding author at pemenend@ucsc.edu.
