# Supplementary material for: The Global Flood Protection Benefits of Mangroves
Source: Sci Rep. 2020 Mar 10;10:4404. doi: 10.1038/s41598-020-61136-6 (PMC7064529; doi:10.1038/s41598-020-61136-6)
Supplement: Supplementary file 1 — Supplementary information. [file 41598_2020_61136_MOESM1_ESM.pdf]

Supplementary material

for

The Global Flood Protection Benefits of Mangroves

Pelayo Menéndez[1,2*], Iñigo J. Losada[1], Saul Torres-Ortega[1], Siddharth Narayan[2], Michael W. Beck[2,3]

1 IHCantabria - Instituto de Hidráulica Ambiental de la Universidad de Cantabria, 39011 Santander, Spain
2 Institute of Marine Sciences, University California, Santa Cruz, CA, 95062 United States of America
3 The Nature Conservancy, Santa Cruz, CA, 950623

*corresponding author: pemenend@ucsc.edu

Supplementary Methods

Baseline case: The Philippines

We used the Philippines results as the baseline case to create a global model and calculate the flood protection service of mangroves worldwide. The large extent of coastlines, complex coastal features, wide presence of mangroves and the large climatic variability, make it an excellent pilot case for valuation of the coastal protection ecosystem service provided by mangroves. Focusing in the climatic variability, the Philippines is at particularly risk from natural hazards like typhoons and regular storms, which are the cause of 80% of the total losses from disasters [average loss totaling nearly 3 billion US$, 29% of this damage is due to coastal flooding (1)]. Another factor that makes the Philippines an interesting country to apply the ecosystem services valuation methodology is the fact that it ranks in the top 15 most habitat-rich countries, with 2,630 km^2 in 2010, representing 2% of the world total (2). However, a constant decline in mangrove extent has been observed in the last few decades. The Philippines has lost 600 km^2 from 1950 to 2010, reducing the original cover from the 3,100 km^2 to 2,500 km^2, that is an average rate of 0.3% yearly loss, much lower than the global observed decline (-1.9% yearly), but nevertheless considerable in terms of mangrove areas lost. Also, the constant pressure on existing mangrove forests from development related land uses like agriculture, aquiculture, urban development or coastal tourism, poses a serious threat to the future of these habitats (3). Mangroves have helped protect The Philippine's coastlines against waves and surges from typhoons and storms in the past (4). This ecosystem also protect the coast against winds (5–7). Some evidences of its protective service were recently observed during Typhoon Haiyan. A study found that across 384 coastal villages found that mangrove presence was significantly correlated with lower death tolls and lower structural damage (8).

Due to these reasons, we valued flood protection service of mangroves in the Philippines by using highly sophisticated numerical model (Delft3D) and modeling both, historical tropical cyclones and regular climate conditions under with and without mangroves scenarios. We made use of these results to upscale globally. Two different results were used for this purpose:

Offshore dynamics generated by tropical cyclones: Offshore waves and storm surge generated by tropical cyclones (IBTrACS database) were numerically simulated by using Delft3D modules "Flow" (9) and "Wave" (10). Both modules were run simultaneously in a 2-dimensional grid of 5 km cell-size with a time step of 30s, forced with hourly wind data and sea level pressure fields obtained from parametric model, in which the non-linear interaction processes of tide, wind setup, inverse barometers and wave setup are considered. The model was validated by comparing the storm surge generated by typhoon Rammasun, in Legaspi and Subic Bay. We use tidal gauges registers from the Global Sea Level Observing System (GLOSS, *http://www.gloss-sealevel.org*) for validation (Supplementary Figure 11).

Waves and surge propagation over the ecosystem, and flood height calculation: Coastal vegetation provides resistance to the energy and flow of waves and water as they come onshore which is modeled by using a friction factor based on Manning coefficient. In the Philippines we classify surface types into three groups: sandy soil (n=0.02) (11), mangroves (n=0.14) (11) and coral reefs (n=0.05) (12). 1-dimensional numerical propagations are carried out using Delft3D model to obtain the flood height along the coast.

Offshore hydrodynamics

Astronomical forcing and sea level: Deep water ocean dynamics have been clearly differentiated between low-intensity events (regular climate) and high-intensity events (tropical cyclones). However, both approaches have some common components, such as the astronomical tide or mean sea level. The astronomical tide is obtained from the global database GOT (Global Ocean Tides), with a resolution of 25 km (ihdata@ihcantabria.com). The historical time series of mean sea level (from 1979 to 2010), show a relevant increase in recent years. The database collects these variations, on a monthly basis, at a scale of 100 km (13).

Tropical cyclones (waves and storm surge): As we did in the Philippines, to address these events on a global scale, we will use the IBTrACS database (Knapp et al., 2010). It contains 6-hour information on tropical storms (winds between 63 km/h and 119 km/h) and tropical cyclones (winds above 119 km/h) in different ocean basins. The greatest cyclonic activity is concentrated in the months of June to November in the northern hemisphere, and between November and March in the southern hemisphere (Supplementary Figure 12). Each tropical storm is characterized by the maximum wind speed and track location at each moment. We analyzed three alternatives to obtain waves and storm surge offshore worldwide: (1) Numerically simulate historical tropical cyclones from the IBTrACS database worldwide using models (e.g. Delft3D), as has been done nationally in the Philippines. (2) Using existing parametric formulations that correlate atmospheric variables (wind and pressure) with oceanographic variables, such as wave height (14) and storm surge

(15). (3) Take advantage of the numerical simulations already carried out with the Delft3D model in the Philippines and look for statistical relationships between the cyclone parameters and the oceanographic variables to create a new predictive model (distance, wind speed, track velocity, wind angle of incidence vs wave height, period, weather tide and duration of the storm peak). The first alternative is not computationally feasible. Although this has been the approach followed in the Philippines, the resources consumed in carrying it out have made it impossible for us to apply it on a global scale. The second option would involve the use of semi-empirical formulas which, in many cases, respond to specific situations and have a very narrow range of applicability. For example, the formula in Ruiz-Martinez et al. (2009) does not predict wave height in the presence of islands, which limits its application to many countries, such as the Philippines. Therefore, we choose the third alternative, based on using the Delft3D model in the Philippines, where 548 events were simulated on a two-dimensional grid of 5 km cell size, finally creating a database of 58 million results. We randomly select the 90% of the generated results to build our predictive model, and keep the other 10% for future validations of the predictive models. We found the correlation between the physical variables of tropical cyclones (distance from the trace to the profile *D* [km], wind speed *W* [km/h], cyclone travel speed *V* [km/h], wind direction from north θ_{WN}, [in degrees] and the angle between the wind direction-profile θ_{WP} [in degrees]) and the oceanographic variables at the target point (maximum significant wave height produced during the event at the target point H_{smax} [in m], peak period *Tp* [in s], maximum storm surge SS_{max} [in m] and maximum storm surge duration, T_{SSmax}). As the predictor variables depend on time, the possible combinations in each tropical cyclone event are infinite, so we must choose significant stationary parameters that accurately predict the ocean climate. The following three alternatives were tested: (1) Tropical cyclone parameters associated to the closest point to each profile, (2) tropical cyclone parameters associated to the maximum wind speed moment and (3) average tropical cyclone parameters. Since the Philippines has an arrangement of islands with coastal areas that are not directly exposed to tropical cyclones, we divide the data into two groups: Coastal areas directly exposed to tropical cyclones and areas protected from the direct impact of tropical cyclones (Supplementary Figure 13). For each combination (5 cyclone variables x 3-time instants x 4 oceanographic variables = 60 cases), we have studied the Pearson coefficient (P_{xy}), which statistically quantifies the degree of correlation between the cyclone variables "X" and the oceanographic variables "Y" (equation S.1). The closer it is to 1 or -1, the greater the dependence on both variables, and, the closer you get to 0, the worse the correlation will be. On the other hand, σ_{xy} is the covariance of the variables 'X' and 'Y', while σ_x and σ_y are the respective standard deviations.

$$P_{xy} = \sigma_{xy} / \sigma_x \cdot \sigma_y \tag{S.1}$$

After studying the cross-correlation between variables, we adjusted ocean climate variables (Yi) to the parametric model of equation S.2. We tested this adjustment for one, two, three and four independent variables (Xi), so that we can cover all the alternatives and, based on the correlation coefficient of each one, choose the best regression model.

$$Y_s = a_0 + a_1 \cdot X_1^{\alpha 1} + a_2 \cdot X_2^{\alpha 2} + \ldots + a_n \cdot X_n^{\alpha n} \tag{S.2}$$

After choosing the minimum values of the Pearson coefficient, the variables that, best predict the maximum wave height (Hs_{max}), the peak period (Tp), the maximum meteorological tide (SS_{max}) and the duration of the meteorological peak produced by the cyclone (Tss_{max}) are shown in Supplementary Table 6. We found different correlations along coastal areas directly exposed (equations S.3 to S.6) than in coastal protected areas (equations S.7 to S.10). In these equations, "D_{min}" is the minimum distance between the storm track and the target point, "W_{dist_min}" is the wind speed when the tropical cyclone is at the closest location to the target point, "W_{mean}" is the average wind speed during along the storm length, "θ_{WN_mean}" is the mean direction of wind respect to the North, "$\theta_{WN\_dist\_min}$" is the wind direction respect to the North at the minimum distance point, "V_{mean}" is the average track velocity, "V_{dist_min}" is the track velocity at the minimum distance point and "V_{wind_max}" is the track velocity at the moment of maximum wind speed.

Regular climate (waves and storm surge): Deep water ocean dynamics produced by any other climate condition different from tropical cyclones is analyzed as regular climate. Wave data comes from the historical series GOW 2 (16), and storm surge data are derived from the reanalysis series created from the DAC (Dynamic Atmospheric Correction) data and from the pressure fields of the 20CR atmospheric reanalysis (NOAA-CIRES, (17)). With the aim of working in the same time interval of both series, the data from 1979 to 2010 have been used. In addition, to avoid double counting of tropical cyclones, records coinciding with these events were removed from the historical wave and storm surge databases used to define the regular climate.

REGRESSION MODEL FOR COASTAL AREAS DIRECTLY EXPOSED TO TROPICAL CYCLONES

$$H_s = 410857 - 0.932 \cdot D_{min}^{0.428} + 3.603e\text{-}154 \cdot \theta_{WP\_dist\_min}^{63.129} - 4.1083 \cdot \theta_{WP\_mean}^{9e\text{-}6} + 0.012 \cdot V_{mean}^{1.115} \quad r=0.90 \tag{S.3}$$

$$T_p = -236.4611 + 0.2781 \cdot D_{min}^{0.406} + 0.2174 \cdot W_{dist\_min}^{0.607} - 38.7856 \cdot \theta_{WN\_mean}^{0.290} + 262.0513 \cdot V_{wind\_max}^{0.090} \quad r=0.90 \tag{S.4}$$

$$SS = 0.2199 - 6.251e\text{-}8 \cdot D_{min}^{2.401} + 3.523e\text{-}42 \cdot W_{dist\_mean}^{4.68} - 7.8412e\text{-}6 \cdot \theta_{WP\_mean}^{2.048} + 4.5341e\text{-}113 \cdot \theta_{WN\_dist\_min}^{46.468} \quad r=0.73 \tag{S.5}$$

$$T_{ss} = -1.6299e6 + 1.6299e6 \cdot W_{mean}^{2e\text{-}5} - 1.1716e\text{-}152 \cdot \theta_{WN\_dist\_min}^{63.133} - 4.5281e\text{-}9 \cdot D_{min}^{3.594} - 2.6318e\text{-}4 \cdot V_{mean}^{2.993} \quad r=0.70 \tag{S.6}$$

REGRESSION MODEL FOR COASTAL AREAS PROTECTED FROM TROPICAL CYCLONES

$$H_s = 9.5249e3 - 1.6819\cdot 10^4\cdot D_{min}^{2e-4} + 7.2955e3\cdot W_{dist\_min}^{5e4} + 0.116\cdot V_{wind\_max}^{0.607} - 5.9172e-60\cdot \theta_{WN\_wind\_max}^{24.13} \quad r=0.92 \quad (S.7)$$

$$T_p = =-5.6228e3 + 0.7901\cdot W_{dist\_min}^{0.43} - 2.531e-154\cdot \theta_{WN\_dist\_min}^{62.955} + 8.4074e-24\cdot D_{min}^{8.982} + 5.6197e3\cdot V_{mean}^{7e-5} \quad r=0.88 \quad (S.8)$$

$$SS = 583.345 - 504.127\cdot D_{min}^{2e-4} + 0.0565\cdot W_{dist\_min}^{0.385} - 78.818\cdot V_{dist\_min}^{8e-4} + 2.978e-108\cdot \theta_{WN\_dist\_min}^{43.89} \quad r=0.83 \quad (S.9)$$

$$T_{ss} = -8.0556\cdot e4 + 4.0666\cdot W_{mean}^{0.6501} + 1.2348e5\cdot D_{min}^{e-4} - 4.2899e4\cdot V_{dist\_min}^{7e-4} + 2.098e152\cdot \theta_{WN\_dist\_min}^{62.699} \quad r=0.88 \quad (S.10)$$

Nearshore hydrodynamics

Tropical cyclones (waves and storm surge): We apply the regression models (equations S.3 to S.10), separately, in each of the 68 regions into which we have divided the world's mangrove coast, and we obtain the same parameters as for regular climate, in addition to the time duration of the meteorological tide (Tss), wave height (Hs), peak period (Tp) and total water level (MSL+AT+SS). Among the 68 regions there are a total of 166 million combinations of tropical cyclones and profiles on which to implement the equations of the estimated regression models.

Regular climate (waves and storm surge): Waves from undefined depths change as they approach the coast and interact with the obstacles they encounter. The combined effect of the processes of shoaling, refraction, diffraction and breakage produce changes in wave height and direction of the fronts. To bring the dynamics of regular weather to the beginning of each profile we have followed a hybrid downscaling. Previously, we have assigned to each profile the closest point of the wave database (GOW 2), meteorological tide (20CR reanalysis), astronomical tide (GOT) and mean sea level. Each time series has a duration of 32 years (from 1979 to 2010), i.e. more than 280,000 hourly sea states to be propagated to each of the more than 700,000 profiles generated worldwide. The only way to deal with such a large number of simulations is to reduce the number of cases to be executed. We applied the MDA algorithm to 3,787 different combinations of historical wave series and levels (AT+SS+MSL). As a result, we went from 3,787 combinations of more than 280,000 sea states to 3,787 combinations of 120 representative sea states. Although there are still many cases, it is possible to approach the calculation using simple models of shoaling and refraction (for wave height) and Snell's law (for direction). We assume that the mean sea level, the storm surge and the astronomical tide are the same offshore and at the seaward side of each profile, so that the total water level (MSL+AT+SS) does not need to be propagated to the profiles. Then, we have, at each of the 700,000 profiles, 120 propagated sea states of wave height (Hs), peak period (Tp), wave direction (θp) and total water level (MSL+AT+SS).

Waves and surge propagation over mangroves

We follow the same strategy to calculate the coastal flood elevation generated by regular weather and generated by tropical cyclones, making use the Philippines´ results, in particular those of the 1-D propagations, run with the Delft3D model. Interpolation tables were created to correlate the climatic information at seaward side of the profile (Hs, Tp, SS and Tss, being this last only specific of tropical cyclones) and the characteristics of the mangroves (width and average depth) with flood height (i.e. total water level along the coast). These tables contain 37,500 tropical cyclone simulations (50 cyclones x 750 profiles) and 90,000 regular climate simulations (120 sea states x 750 profiles), that aims to be extrapolated at global scale (see Supplementary Figure 8 and Supplementary Figure 9). When interpolating in the two tables, we obtain the flood height in each profile, both for regular climate and tropical cyclones, but with the following particularities: In the case of regular climate, we interpolate 120 sea states representative of the 32-year time series. Therefore, we must apply the statistical reconstruction technique RBF (Radial Basis Functions) (18) to reconstruct in each profile the complete historical flood height series. In the case of tropical cyclones, we interpolate all the events that impact each profile (unclassified), so no reconstruction is necessary. Then we select the maximum values on a variable threshold, so that, at least, we assure 1 event every 5 years. We adjust these selected values to a Generalized Pareto-Poisson distribution and obtain the flood elevation return period curves for each mangrove conservation scenario.

Flood modelling process

To transfer the flood height into land, we use bathtub method. In order to be consistent in the analysis of both climatic conditions, we chose the same return periods with which to obtain the flood height: 10, 25, 50 and 100 years. The minimum incidence rate is marked by the limitation of some areas with a shortage of extreme events, where there are no records of frequent flooding. We use the GIS "bathtub" method to connect the points of the global SRTM topography (30 meters horizontal resolution) that are below the water level. Supplementary Figure 14 is an example of this fourth step of the methodology. It shows 1-in-25-year coastal flooding produced by tropical cyclones in Cancun (Mexico). Both scenarios, with and without mangrove are represented in the figure.

Calculating people and property flooded

The assessment of the consequences of the loss of mangroves will be made in terms of people and property damage. However, instead of estimating the property value on the basis of national economic indicators, we will calculate it directly as the sum of two spatial distributions of stock (industrial and residential), for which we do have global information. The spatial distribution of people used is the GPW, from SEDAC, with a resolution of 1 km. On the other hand, the spatial distribution of residential and industrial stock that we have used comes from the GAR15 database and is at a scale of 5 km of spatial resolution throughout the world. Intersecting flood layer with people and stock layers requires to redistribute the assets from 1 km and 5 km (respectively) over a mesh of 30 meters resolution.
The sensitivity of people and stock to different levels of flooding was obtained through different damage functions. Damage functions provide information of the number of people affected by coastal flooding and the stock losses, according to the water depth. We use different damage functions for population and for stock: Population damage is based on the hypothesis than water depths below 0.5 meters do not affect people, while water depths above 0.5 meters affect 100% of people hit by flooding. It is a common practice in the scientific literature not to use damage functions to calculate the population affected by floods(19). This option overestimates the results obtained; therefore, it is recommended to opt for a certain threshold below which the effects of flooding are not considered (20). This threshold is set at 0.5 meters because it is a common value used by emergency services (Japan, Netherlands, US) in determining whether or not it is necessary to evacuate people from an area under threat. In case of stock, we adapted the "Global Flood Depth-Damage Functions" from Huizinga/JRC (Joint Research Centre) broken down by continent (Africa, Asia, Oceania, North America, South America and Central America) and by asset type: residential and industrial (21).
The combination of damage curves and the distribution of people and stock exposed to flooding gives us the consequences per return period. All that remains now is to annualize the damages and express them in terms of Expected Annual Values.

Assessing Expected Annual Damages and Benefits

The expected annual damage is calculated by the integration of the flood damage density curve over all probabilities. We deal with limited data associated to different return periods and, consequently, to accurately compute expected annual damages we must increase the amount of probability points by fitting data to the best-fit equation. We tested two possibilities to fit people and property damages: power law (Eq. S.11 in bold) and logarithmic law (Eq. S.12 in bold). In both equations, "Y" is the damage [$D(T_r)$ or $D(p)$] associated to 1-in-X-year event (T_r). The return period "X" could be also represented as the inverse probability (T_r=1/p). Coefficients "a" and "b" (Eq. S.11) and "c" and "d" (Eq. S.12) are the best fit parameters of the regression analysis.

$Y=a\cdot X^b$ → $D(T_r) = a\,(T_r)^b$ → $D(p) = a\,(1/p)^b$ Power law (S.11)

$Y=c\cdot \ln X+d$ → $D(T_r) = c \ln (T_r) + d$ → $D(p) = c \ln (1/p) + d$ Logarithmic law (S.12)

The integration could be carried out by using numerically (Eq. S.13), by using the trapezoidal rule, or analytically (Eq. S.14). As we had previously obtained two possible analytical equations that best fit data, we solved the Expected Annual Damaged following the analytical solution (Eq. S.14).

$$EAD = 0.5 \sum_{i=1}^{i=length(Tr)} \left(\frac{1}{Tr_i} - \frac{1}{Tr_{i+1}}\right)(D_i + D_{i+1}) \quad (S.13)$$

$$EAD=\int_{p=\frac{1}{Tr_{MAX}}}^{p=\frac{1}{Tr_{min}}} D(p)\, dp \quad (S.14)$$

It is not possible to calculate damage for all the probabilities, from p=0 (Tr=∞) to p=∞ (Tr=0), forcing us to choose the maximum and minimum return period to truncate the calculation of Expected Annual Damage. We substitute the previous best fit equations (Eq. S.11 and S.4) in Eq. S.14 and we analytically solve the integral, to obtain the following (Eq. S.15 and S.16):

$$AED = \int_{p=\frac{1}{Tr_{MAX}}}^{p=\frac{1}{Tr_{MIN}}} a\left(\frac{1}{p}\right)^b dp = \left[a\frac{p^{-b+1}}{-b+1}\right]_{p=\frac{1}{Tr_{MAX}}}^{p=\frac{1}{Tr_{MIN}}} \quad \text{Power law} \quad (S.15)$$

$$AED = \int_{p=\frac{1}{Tr_{MAX}}}^{p=\frac{1}{Tr_{MIN}}} d + c\ln\left(\frac{1}{p}\right) dp = [d\cdot p + c\cdot p - c\cdot p\cdot \ln(p)]_{p=\frac{1}{Tr_{MAX}}}^{p=\frac{1}{Tr_{MIN}}} \quad \text{Logarithmic law} \quad (S.16)$$

Power law is better correlated with data than the logarithmic law (Supplementary Table 7). The average determination coefficient (r^2) of the power law is higher than the one of the potential law (r^2~0.97 vs r^2~0.94). Then, final results of Expected Annual Damages will be obtained from Eq. S.15. However, we still need to truncate the values of Tr_{min} and Tr_{max}. The EAD is very sensitive to the minimum value of return period selected Tr_{min} (Supplementary Figure 15), but

not to the maximum (Tr_{max}) when it exceeds a certain return period (Supplementary Figure 16). We first fix Tr_{max}=100 year (the real maximum return period from the original datasets) and we test Tr_{min}, from 1 to 10 years (Supplementary Figure 15). Then, we secondly fix Tr_{min}=5 year (the real minimum return period from the original datasets) and we test Tr_{max} from 20 to 1,000 years (Supplementary Figure 16). With this analysis we can confirm that computing return periods larger than 1-in-100-year events do not significantly change the EAD.

Assumptions: Model validation and sensitivity analysis

All the assumptions made along this work are addressed at . The validation process was developed with the aim of covering several issues: (1) validate the capacity of the numerical models used in the Philippines for the simulation of waves and surge generated by tropical cyclones. (2) Validate the modeling strategy waves and surge propagation over mangroves using cross-shore profiles. (3) Validate the regression models obtained to estimate the offshore hydrodynamics. In addition, we studied the sensitivity of Delft3D to changes in hydrodynamic conditions, mangrove characteristics and the combined effect of the of other ecosystems, such as coral reefs.

Offshore validation in the Philippines: Delft3D model was validated in the Philippines by comparing the Total Water Level and the storm surge produced by one single event, Typhoon Rammasun (July 2014) with historical instrumental data at buoys located in Legaspi and Subic Bay (Supplementary Figure 11). Different forcing methods were tested combining wind, waves, storm surge and astronomical tide conditions (22). The good agreement observed in the comparison of numerical results with instrumental data shows high capacity of the model to reproduce sea level and waves induced by tropical cyclones. The validation tests also demonstrated that swell waves do not modify the storm surge due to the low intensity of the swell component in The Philippines with respect to wind components (22).

Nearshore validation in the Philippines: Nearshore waves and storm surge validation was carried out by comparing the induced Total Water Level obtained with both, 1-dimensional and 2-dimensional numerical simulations (Delft3D) and the instrumental data measured in Pagbilao bay during typhoon Betty (category 5) which hit the Philippines from 7th to 17th august 1987. The 2-dimensional case was forced with IBTrACS winds in a 100-meter resolution meshgrid covering Pagbilao bay. Meanwhile, the 1-dimensional model was forced with offshore waves and storm surge propagated over 100-meter spaced transects. flood height obtained in both cases overestimate the field data because the 100-meter resolution mesh do not have the capacity to capture the effect of mangroves. Smaller cell size, of the order of 10 meters, is required to be able to simulate wave and sea level propagation in mangrove areas.

Tropical cyclones regression model validation: To justify the need to create our own regression models, instead of using some of the existing ones in the literature, we have compared the adjustments applied in this study (equations S.3 to S.10) with the maximum wave height and peak period formulations of Ruiz-Martinez et al. (2009) and Ochi (1993). We applied both methods to a specific event in the Philippines, drawing the following conclusions:

On the one hand, Ruiz-Martinez model provides results very similar to those of our regression model for areas directly exposed to tropical cyclones. Both, the maximum wave height best fit equation (equation S.3) and the peak period adjustment (equation S.4) are no more than 15% different from the estimates of Ruiz-Martinez (2009), as seen in Supplementary Figure 17. On the other hand, Ochi model provides results very similar to those obtained with our regression model of cyclone protected areas, but only for maximum wave height (equation S.7), as can be seen in the left panel of Supplementary Figure 18. The peak period in protected areas differs to our model (equation S.8), and the differences in these coastal areas that are not in direct exposure to cyclones are in the order of 30%-50% (right panel, Supplementary Figure 18). These observations highlight the need to separate the directly exposed areas from those that are not, with the aim of accurately representing the maritime climate generated by tropical cyclones in any coastal area.

Sensitivity analysis nº1 (Storm surge intensity, storm surge duration and mangroves length): A set of theoretical simulations of a storm-surge event on a 1D grid of 5001 cells of 5 m sides were developed by propagating different storm conditions (0.5, 1.2 and 3 m of storm surge height and 2, 4, 6 and 8 hours of storm surge duration) over eight mangroves extensions (0, 2, 4, 6, 8, 10, 12 and 15 km) with a fixed roughness coefficient of 0.15 over the habitat and 0.02 at any other bottom transect. After 96 simulations the main conclusions obtained were that (1) for the same storm surge, longer pulse durations and lower friction result in more flooding, (2) Storm surge duration is the most critical variable affecting flooding level and (3) Larger mangroves decrease water level (dissipation) and, consequently, the flooding extension.

Sensitivity analysis nº2 (combined effect of the presence of both, mangroves and coral reefs): To ascertain how the Delft3D model can provide the Total Water Level for both, coral reefs and mangroves combined effect, four numerical tests comparing scenarios only with mangroves, only with coral reefs, without any of the habitats and with both ecosystems were carried out. Results show that the presence of both coral reefs and mangroves provide more than a 149% reduction in flood height as compared to the case of absence of vegetation. Furthermore, mangroves provide itself more than a 102% reduction in flood height, while coral reefs only reduced 8% the coastal water level (Supplementary Figure 19). The results derived from the sensitivity analysis concluded that mangroves contribute significantly to storm surge reduction; while reefs are effective submerged defenses to dissipate waves by means of increasing breaking processes and bottom friction. It is essential to consider the combined effect of the presence of corals and mangroves because this situation is found along 37% of the Philippine coast, being the most common combination. In addition, 63% of the mangroves are protected with coral reefs located in front of them.

Sensitivity analysis nº3 (effect of Manning coefficient): We tested the effect of land cover into the model by varying Manning's coefficient (n) with the aim of improving the accuracy of flood height calculation. The sensitivity analysis was carried out considering open water Manning coefficient (n=0.02) except in mangrove areas, where we set n=0.14. We tested all the cross-shore profiles for all different waves and surge conditions, and we obtained that, as an average, every single decrease of Manning Coefficient (Δn= -0.01) results in a 6.6% more of flood height Supplementary Figure 20.

References Supplementary material

1. National Economic and Development Authority. Philippine Development Plan 2017-2022. 2017;1–452. Available from: http://pdp.neda.gov.ph/wp-content/uploads/2017/01/PDP-2017-2022-05-11-2017.pdf
2. Giri C, Ochieng E, Tieszen LL, Zhu Z, Singh A, Loveland T, et al. Status and distribution of mangrove forests of the world using earth observation satellite data. Glob Ecol Biogeogr. 2011;20(1):154–9.
3. Giri C, Long J, Abbas S, Murali RM, Qamer FM, Pengra B, et al. Distribution and dynamics of mangrove forests of South Asia. J Environ Manage [Internet]. 2015;148:101–11. Available from: http://dx.doi.org/10.1016/j.jenvman.2014.01.020
4. Bricker JD, Takagi H, Mas E, Kure S, Adriano B, Yi C, et al. Spatial variation of damage due to storm surge and waves during Typhoon Haiyan in the Philippines. Publ Japan Soc Civ Eng B2 (Coastal Eng. 2014;70(2):I_231-I_235.
5. Walters BB. Local Mangrove Planting in the Philippines : Are Fisherfolk and Fishpond Owners Effective Restorationists ? Restor Ecol. 2000;8(3):237–46.
6. Walters BB. Local Management of Mangrove Forests in the Philippines : Successful Conservation or Efficient Resource Exploitation ? Hum Ecol. 2004;32(2):177–95.
7. Williams MJ, Coles R, Primavera JH. A lesson from cyclone Larry : An untold story of the success of good coastal planning. 2007;71:364–7.
8. Seriño MN, Ureta JC, Baldesco J, Galvez KJ, Predo C, Seriño EK. Valuing the Protection Service Provided by Mangroves in Typhoon-hit Areas in the Philippines [Internet]. Baybay City (Philippines); 2017. Available from: http://www.eepsea.org/pub/rr/2017-RR19Serino_web.pdf
9. Delft3D-FLOW user manual. Delft, the Netherlands. 2006;
10. Delft3D-WAVE user manual. Delft, the Netherlands. 2000;
11. Zhang K, Liu H, Li Y, Xu H, Shen J, Rhome J, et al. The role of mangroves in attenuating storm surges. Estuar Coast Shelf Sci [Internet]. 2012;102–103:11–23. Available from: http://dx.doi.org/10.1016/j.ecss.2012.02.021
12. Prager EJ. Numerical simulation of circulation in a Caribbean-type backreef lagoon - A preliminary study. Coral Reefs. 1991;10(4):177–82.
13. Church JA, White NJ, Coleman R, Lambeck K, Mitrovica JX. Estimates of the regional distribution of sea level rise over the 1950-2000 period. J Clim. 2004;17(13):2609–25.
14. Ruiz-Martinez G, Silva-Casarín R, Pérez-Romero DM, Posada-Vanegas G, Bautista-Godínez EG. Hybrid model for ocean wave characterization | Modelo híbrido para la caracterización del oleaje. Ing Hidraul en Mex. 2009;24(3):5–22.
15. Dean RG, Dalrymple RA. Water wave mechanics for engineers and scientists. Vol. 2. world scientific publishing Co Inc; 1991.
16. Perez J, Menendez M, Losada IJ. GOW2: A global wave hindcast for coastal applications. Coast Eng [Internet]. 2017;124(April):1–11. Available from: http://dx.doi.org/10.1016/j.coastaleng.2017.03.005
17. Compo GP, Whitaker JS, Sardeshmukh PD, Matsui N, Allan RJ, Yin X, et al. The Twentieth Century Reanalysis Project. Q J R Meteorol Soc. 2011;137(654):1–28.
18. Camus P, Mendez FJ, Medina R, Tomas A, Izaguirre C. High resolution downscaled ocean waves (DOW) reanalysis in coastal areas. Coast Eng [Internet]. 2013;72:56–68. Available from: http://dx.doi.org/10.1016/j.coastaleng.2012.09.002
19. Hinkel J, Lincke D, Vafeidis AT, Perrette M, Nicholls RJ, Tol RSJ, et al. Coastal flood damage and adaptation costs under 21st century sea-level rise. Proc Natl Acad Sci [Internet]. 2014;111(9):3292–7. Available from: http://www.pnas.org/lookup/doi/10.1073/pnas.1222469111
20. Hallegatte S, Green C, Nicholls RJ, Corfee-Morlot J. Future flood losses in major coastal cities. Nat Clim Chang [Internet]. 2013;3(9):802–6. Available from: http://dx.doi.org/10.1038/nclimate1979
21. Huizinga J, De Moel H, Szewczyk W. Global flood depth-damage functions - Methodology and the database with guidelines [Internet]. 2017. 108 p. Available from: http://publications.jrc.ec.europa.eu/repository/bitstream/JRC105688/global_flood_depth-damage_functions__10042017.pdf
22. Menéndez P, Losada IJ, Beck MW, Torres-Ortega S, Espejo A, Narayan S, et al. Valuing the protection services of mangroves at national scale: The Philippines. Ecosyst Serv. 2018;34(Part A):24–36.
23. Knapp KR, Kruk MC, Levinson DH, Diamond HJ, Neumann CJ. The international best track archive for climate stewardship (IBTrACS) unifying tropical cyclone data. Bull Am Meteorol Soc. 2010;91(3):363–76.
24. Cid A, Camus P, Castanedo S, Méndez FJ, Medina R. Global reconstructed daily surge levels from the 20th Century Reanalysis (1871–2010). Glob Planet Change [Internet]. 2017;148:9–21. Available from: http://dx.doi.org/10.1016/j.gloplacha.2016.11.006
25. Beck MW, Narayan S, Trespalacios D, Pfliegner K, Losada IJ, Menéndez P, et al. The Global Value of

Mangroves for Risk Reduction. Summary Report. Berlin; 2018.
26. Beck MW, Losada IJ, Menéndez P, Reguero BG, Díaz-Simal P, Fernández F. The global flood protection savings provided by coral reefs. Nat Commun. 2018;9(1).
27. Camus P, Mendez FJ, Medina R, Cofiño AS. Analysis of clustering and selection algorithms for the study of multivariate wave climate. Coast Eng. 2011;58(6):453–62.
28. Camus P, Mendez FJ, Medina R. A hybrid efficient method to downscale wave climate to coastal areas. Coast Eng [Internet]. 2011;58(9):851–62. Available from: http://dx.doi.org/10.1016/j.coastaleng.2011.05.007
29. Reguero BG, Menéndez M, Méndez FJ, Mínguez R, Losada IJ. A Global Ocean Wave (GOW) calibrated reanalysis from 1948 onwards. Coast Eng [Internet]. 2012;65:38–55. Available from: http://dx.doi.org/10.1016/j.coastaleng.2012.03.003
30. Guanche Y, Camus P, Guanche R, Mendez FJ, Medina R. A simplified method to downscale wave dynamics on vertical breakwaters. Coast Eng. 2013;71:68–77.
31. Menéndez P, Losada IJ, Beck MW, Torres-Ortega S, Espejo A, Narayan S, et al. Valuing the protection services of mangroves at national scale: The Philippines. Ecosyst Serv. 2018;34.
32. Yamazaki D, Ikeshima D, Tawatari R, Yamaguchi T, O'Loughlin F, Neal JC, et al. A high-accuracy map of global terrain elevations. Geophys Res Lett. 2017;44(11):5844–53.
33. Bono A De, Mora MG. International Journal of Disaster Risk Reduction A global exposure model for disaster risk assessment. Int J Disaster Risk Reduct [Internet]. 2014;10:442–51. Available from: http://dx.doi.org/10.1016/j.ijdrr.2014.05.008

Supplementary Figures

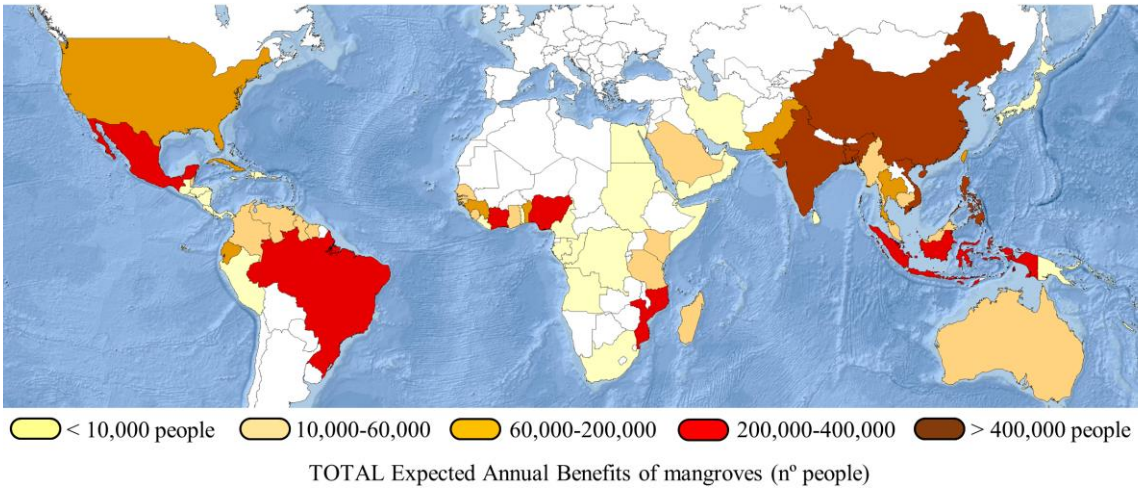

Supplementary Figure 1: Country distribution of total expected annual benefits (tropical cyclones and regular climate) to people (nº of people). *Base maps reprinted from ArcGIS Online maps under a CC BY license, with permission from Esri, original Copyright © 2018 Esri (Basemaps supported by Esri, DigitalGlobe, GeoEye, Earthstar Geographics, CNES/Airbus Ds, USDA, AEX, Getmapping, Aerogrid, IGN, IGP, swisstopo, and the GIS User Community).*

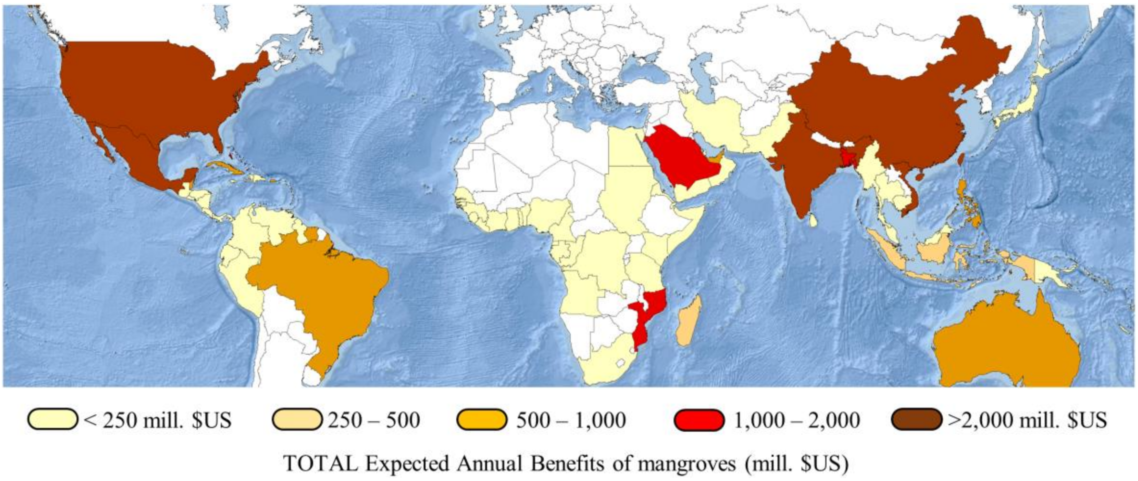

Supplementary Figure 2: Country distribution of total expected annual benefits (tropical cyclones and regular climate) to property ($US million). *Base maps reprinted from ArcGIS Online maps under a CC BY license, with permission from Esri, original Copyright © 2018 Esri (Basemaps supported by Esri, DigitalGlobe, GeoEye, Earthstar Geographics, CNES/Airbus Ds, USDA, AEX, Getmapping, Aerogrid, IGN, IGP, swisstopo, and the GIS User Community).*

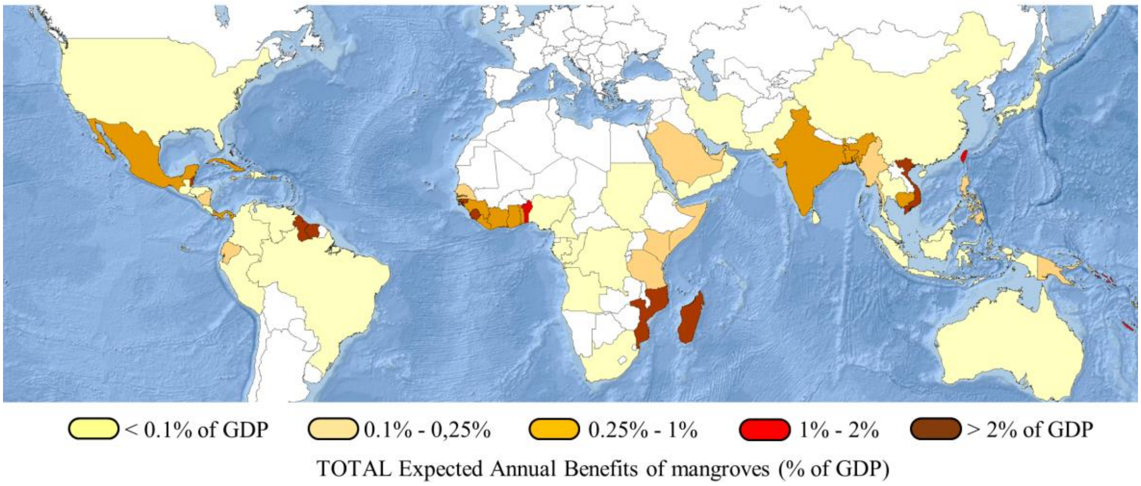

Supplementary Figure 3: Country distribution of total expected annual benefits (tropical cyclones and regular climate) to property, relative to the national GDP (%). *Base maps reprinted from ArcGIS Online maps under a CC BY license, with permission from Esri, original Copyright © 2018 Esri (Basemaps supported by Esri, DigitalGlobe, GeoEye, Earthstar Geographics, CNES/Airbus Ds, USDA, AEX, Getmapping, Aerogrid, IGN, IGP, swisstopo, and the GIS User Community).*

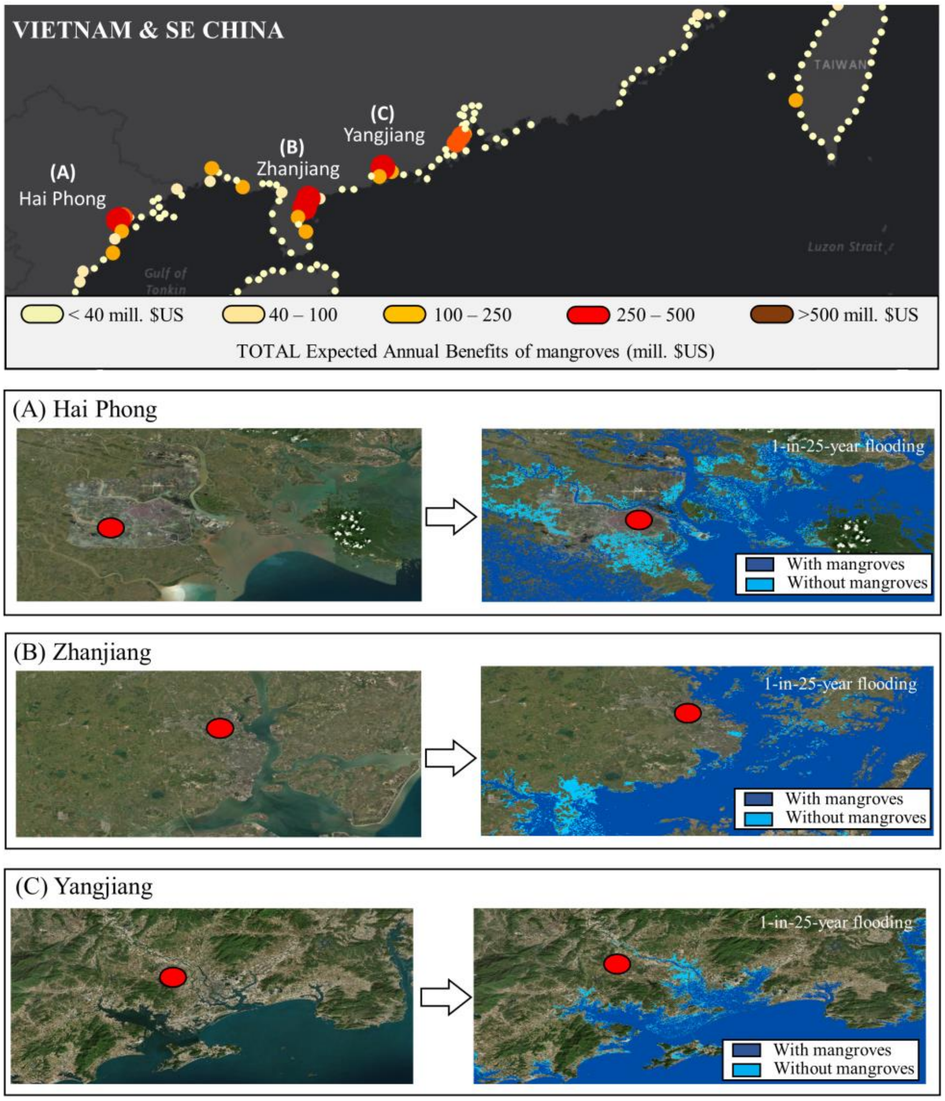

Supplementary Figure 4: Annual Expected Property Benefits provided by mangroves in north Vietnam and Southeast China coast. *Panels (A), (B) and (C) show the flood maps of 1-in-25-year tropical cyclone induced flooding event in three key coastal areas. Figure created with ArcMap | ArcGIS Desktop software (10.7.1 version, http://desktop.arcgis.com/en/arcmap/). Online maps under a CC BY license, with permission from Esri, original Copyright 2018 Esri (Basemaps supported by Esri, DigitalGlobe, GeoEye, Earthstar Geographics, CNES/Airbus Ds, USDA, AEX, Getmapping, Aerogrid, IGN, IGP, swisstopo, and the GIS User Community).*

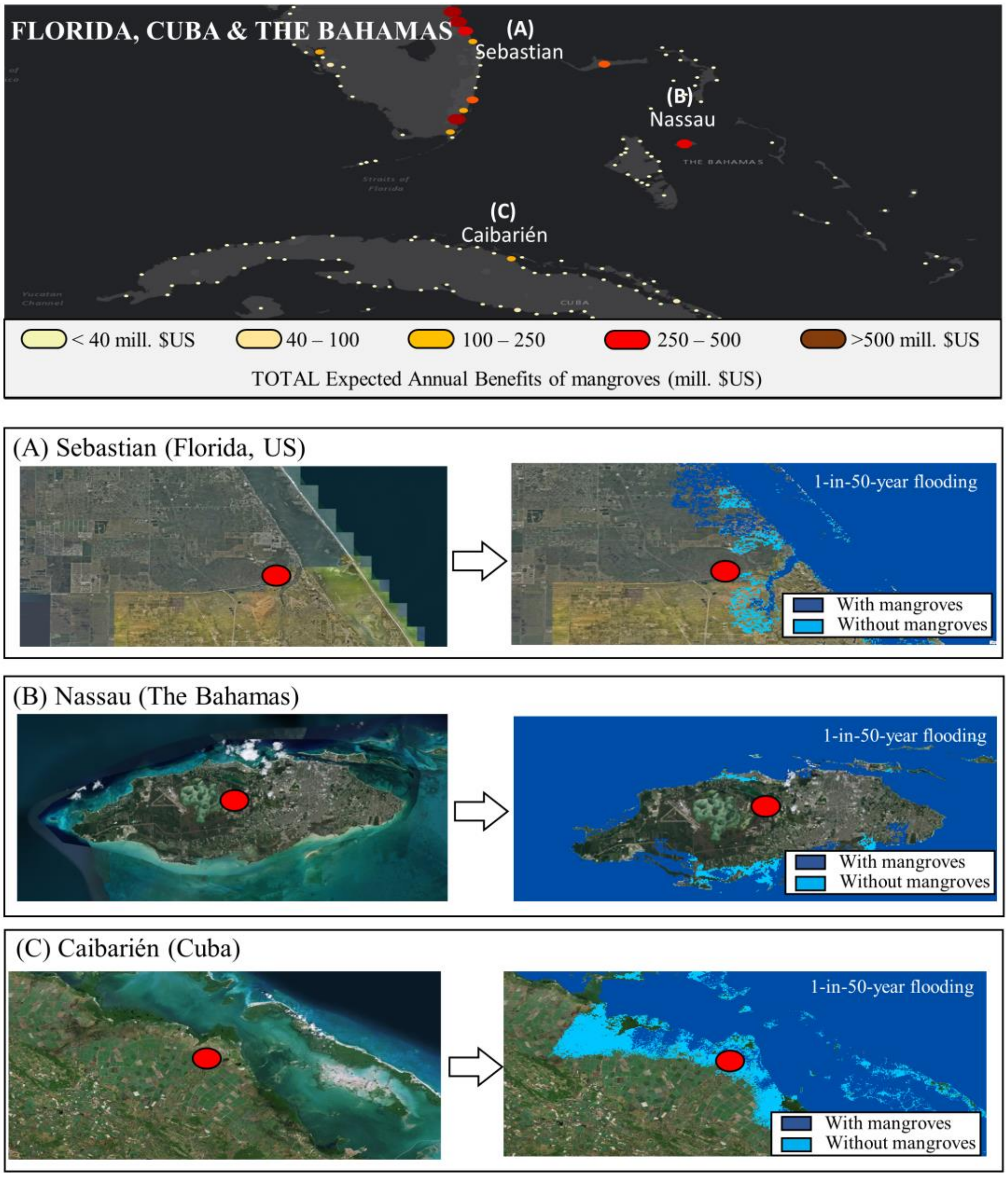

Supplementary Figure 5: Annual Expected Property Benefits provided by mangroves in North Caribbean (Florida, The Bahamas and Cuba). *Panels (A), (B) and (C) show the flood maps of 1-in-50-year tropical cyclone induced flooding event in three key coastal areas. Figure created with ArcMap | ArcGIS Desktop software (10.7.1 version, http://desktop.arcgis.com/en/arcmap/). Online maps under a CC BY license, with permission from Esri, original Copyright 2018 Esri (Basemaps supported by Esri, DigitalGlobe, GeoEye, Earthstar Geographics, CNES/Airbus Ds, USDA, AEX, Getmapping, Aerogrid, IGN, IGP, swisstopo, and the GIS User Community).*

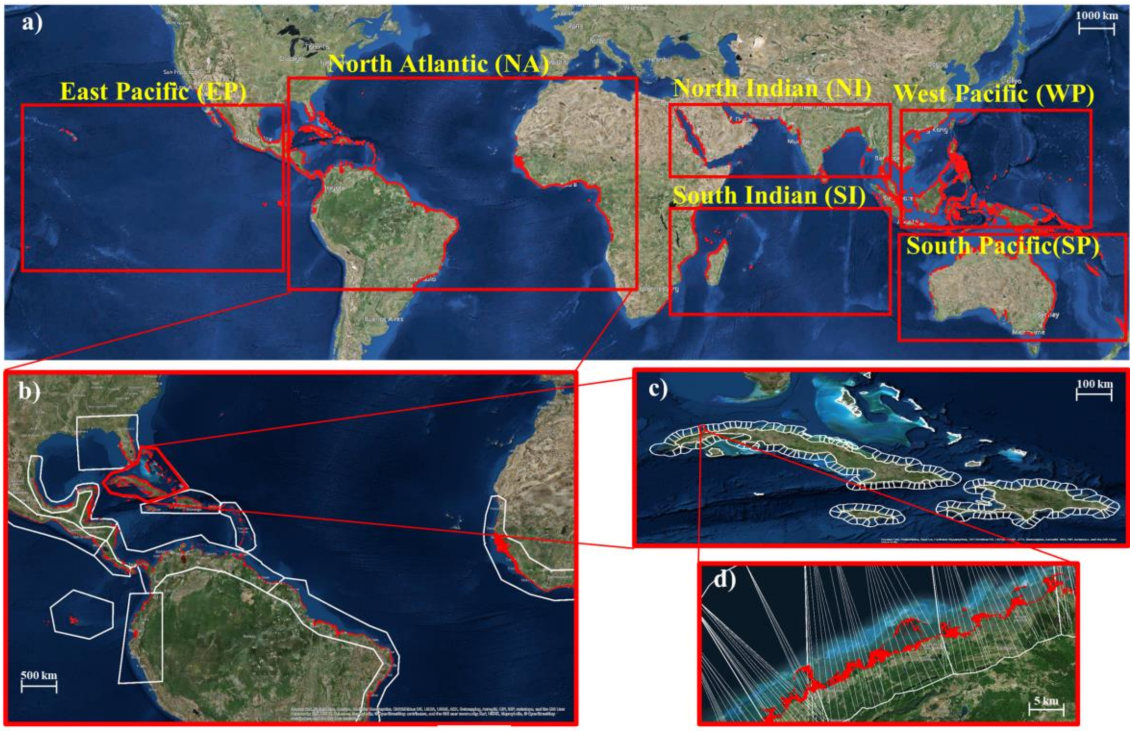

Supplementary Figure 6: Different world subdivision level to address the study of coastal flood protection of mangroves. *(a) Macro-regions with the global mangrove cover in red, (b) Sub-regions in the Atlantic Ocean basin, (c) Local study units every 20 km of coastline in the Northern Caribbean Sub-region, (d) Profiles every 1 km of coastline in the North of Cuba. Figure created with ArcMap | ArcGIS Desktop software (10.7.1 version, http://desktop.arcgis.com/en/arcmap/). Online maps under a CC BY license, with permission from Esri, original Copyright 2018 Esri (Basemaps supported by Esri, DigitalGlobe, GeoEye, Earthstar Geographics, CNES/Airbus Ds, USDA, AEX, Getmapping, Aerogrid, IGN, IGP, swisstopo, and the GIS User Community).*

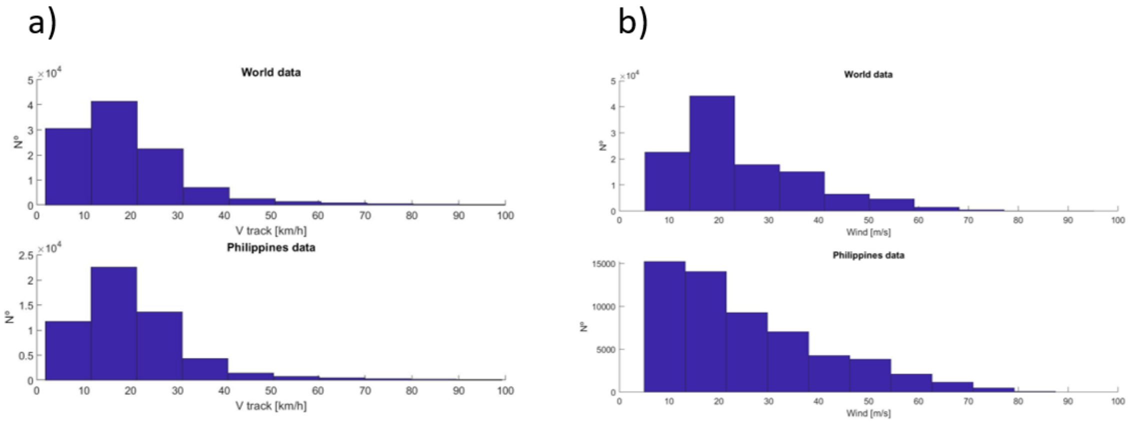

Supplementary Figure 7: Occurrence histogram of the storm parameters. *(a) Velocity track and (b) maximum wind speed in the Philippines (lower panels) and worldwide (upper panel).*

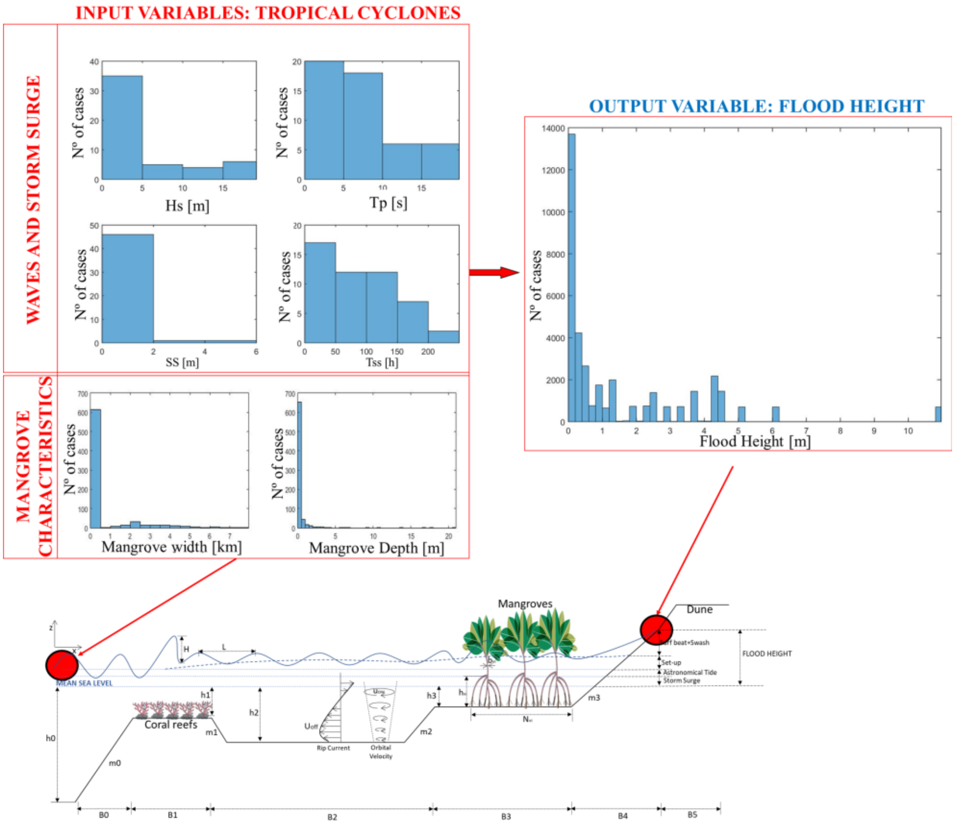

Supplementary Figure 8: Range of the interpolation parameters used to estimate the flood height generated by tropical cyclones. *Self-elaboration using Microsoft PowerPoint software (2018 version, https://office.microsoft.com) and Matlab software (2019a version, https://www.mathworks.com/products/matlab.html)*

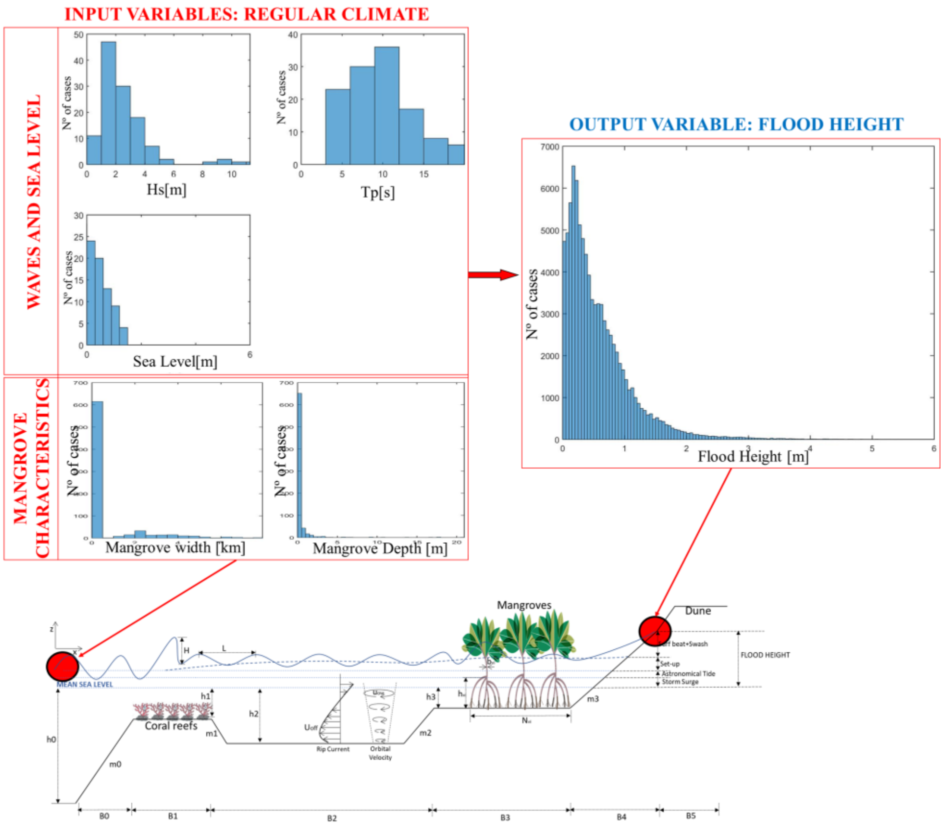

Supplementary Figure 9: Range of the interpolation parameters used to estimate the flood height generated by regular climate. *Self-elaboration using Microsoft PowerPoint software (2018 version, https://office.microsoft.com) and Matlab software (2019a version, https://www.mathworks.com/products/matlab.html)*

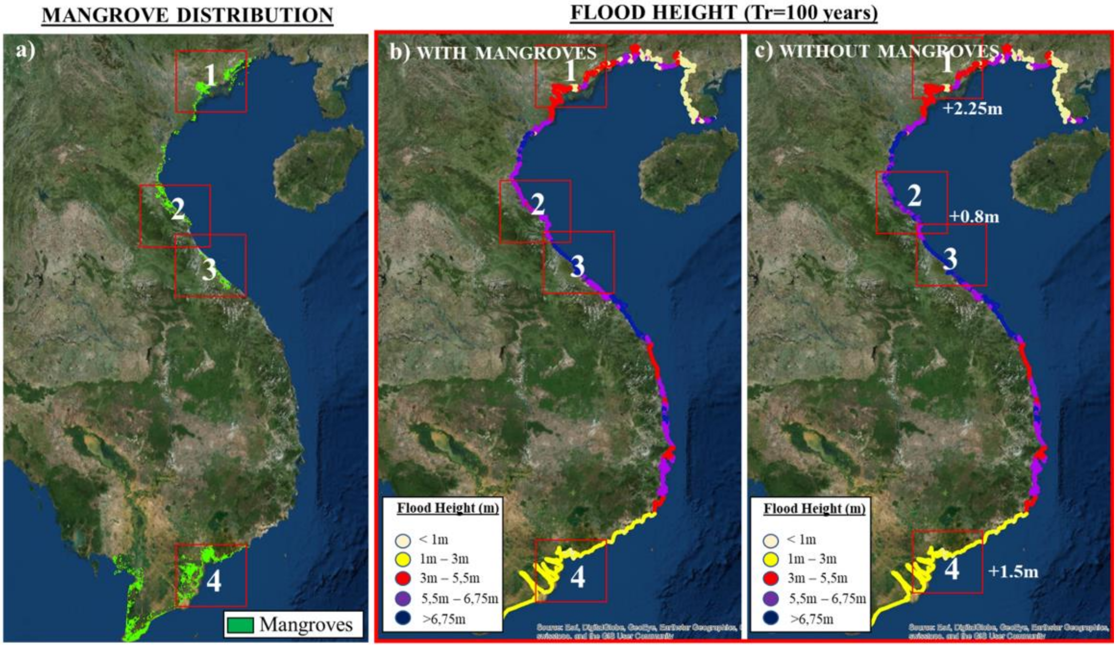

Supplementary Figure 10: Example of a 1-in-100-year flood height (FH) produced by tropical cyclones along the coast of Vietnam. *Two scenarios: with mangroves (b) and without mangroves (c). Image (a) shows the ecosystem distribution along the country's coast (in green). The four white squared areas correspond to places with the highest ecosystem density, although not all provide the same level of protection. While the mangroves in zones 2 and 3 do little to reduce flood levels, those in zones 1 and 4 manage to reduce the level more than 1 m. Figure created with ArcMap | ArcGIS Desktop software (10.7.1 version, http://desktop.arcgis.com/en/arcmap/). Online maps under a CC BY license, with permission from Esri, original Copyright 2018 Esri (Basemaps supported by Esri, DigitalGlobe, GeoEye, Earthstar Geographics, CNES/Airbus Ds, USDA, AEX, Getmapping, Aerogrid, IGN, IGP, swisstopo, and the GIS User Community).*

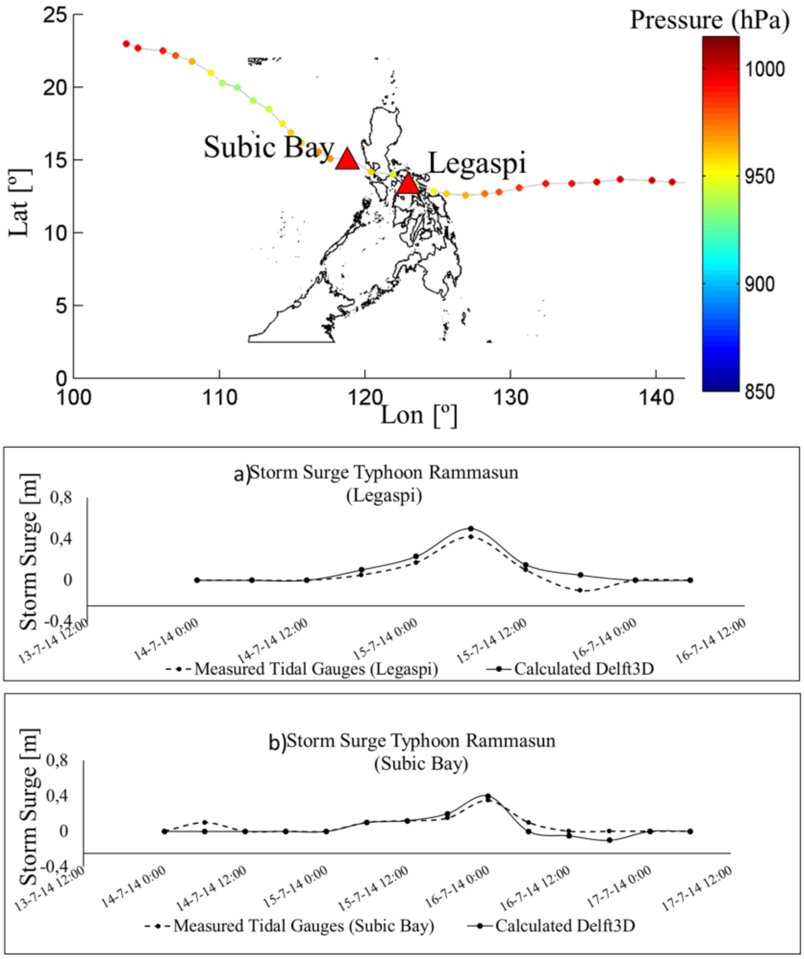

Supplementary Figure 11: Validation of the simulated storm surge (in meters) of Typhoon Rammasun in Legaspi and Subic Bay tide gauges. *The upper panel represent the cyclone track with the minimum pressure in hPa. The lower panels (a) and (b) represent the comparison between the predicted storm surge (using Delft3D model) and the field measurements.*

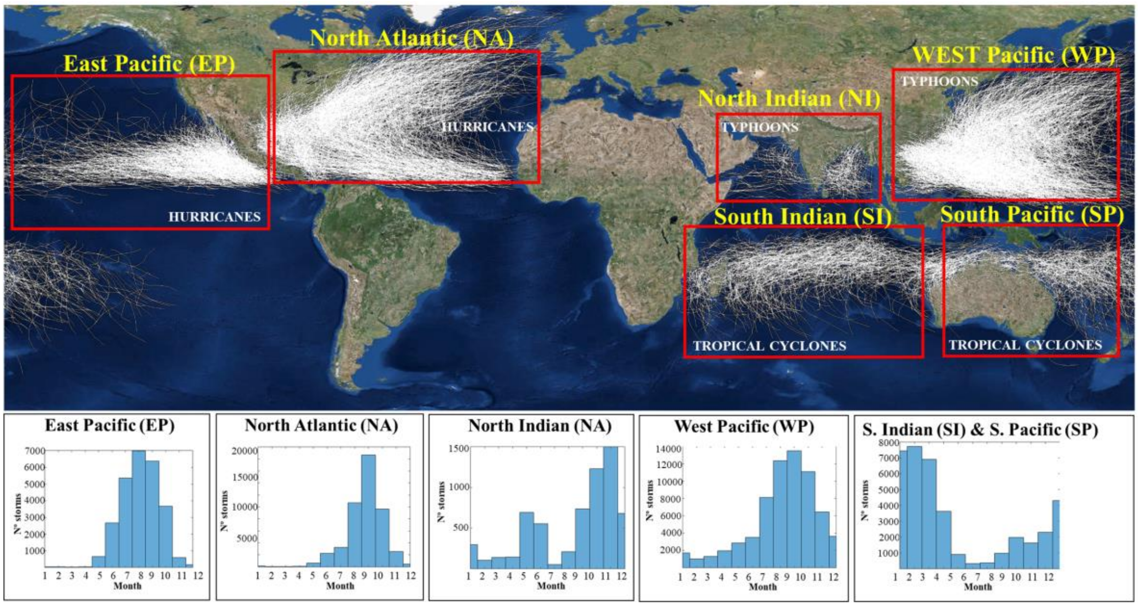

Supplementary Figure 12: IBTrACS data divided by ocean basins (upper map), and annual distribution histograms of the number of cyclones (lower graphs). *Figure created with ArcMap | ArcGIS Desktop software (10.7.1 version, http://desktop.arcgis.com/en/arcmap/). Online maps under a CC BY license, with permission from Esri, original Copyright 2018 Esri (Basemaps supported by Esri, DigitalGlobe, GeoEye, Earthstar Geographics, CNES/Airbus Ds, USDA, AEX, Getmapping, Aerogrid, IGN, IGP, swisstopo, and the GIS User Community).*

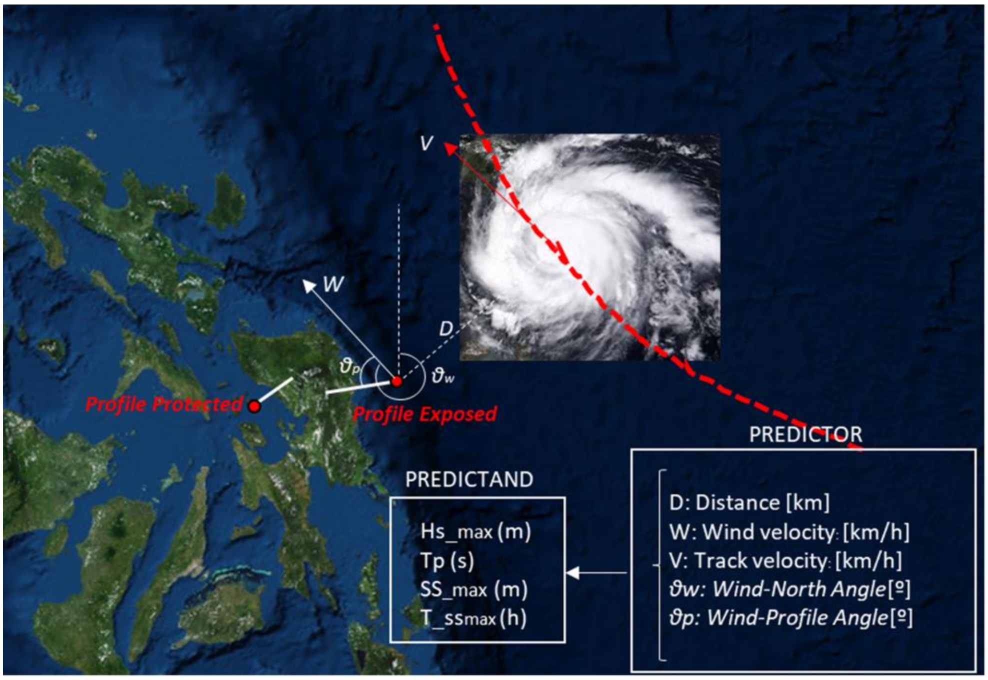

Supplementary Figure 13: Sketch to identify the predictor variables of the maritime climate generated by a tropical cyclone at any point. *The figure shows the example of a hurricane in the Philippines where the predictor variables are the distance from the eye of the hurricane to the target point (D), the wind speed (W), the speed of displacement of the cyclone (V), the wind direction relative to the North at the target point (θw) and the angle between the wind direction and the direction of the profile linking the target point and the coast (θp). The predicting climatic variables are the maximum significant wave height produced during the event at the target point (H_{smax}), the peak period (Tp), the maximum weather tide (SS_{max}) and the maximum weather tide duration (T_{SSmax}). Figure created with ArcMap | ArcGIS Desktop software (10.7.1 version, http://desktop.arcgis.com/en/arcmap/). Online maps under a CC BY license, with permission from Esri, original Copyright 2018 Esri (Basemaps supported by Esri, DigitalGlobe, GeoEye, Earthstar Geographics, CNES/Airbus Ds, USDA, AEX, Getmapping, Aerogrid, IGN, IGP, swisstopo, and the GIS User Community).*

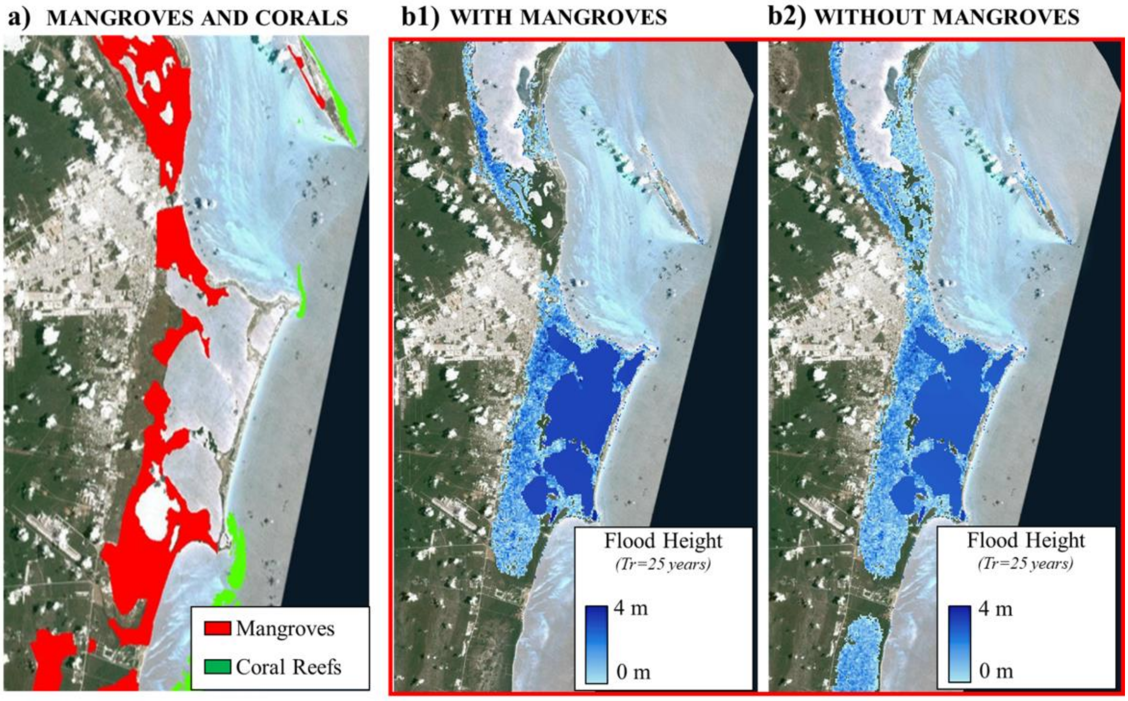

Supplementary Figure 14: 1-in-25-year flood area in Cancun due to tropical cyclones. *(a) Distribution of the mangrove (red) and coral (green) layer in this case study area. (b) Flood mask produced by tropical cyclones with current mangrove cover. (c) Flood mask produced by tropical cyclones without mangroves. Figure created with ArcMap | ArcGIS Desktop software (10.7.1 version, http://desktop.arcgis.com/en/arcmap/). Online maps under a CC BY license, with permission from Esri, original Copyright 2018 Esri (Basemaps supported by Esri, DigitalGlobe, GeoEye, Earthstar Geographics, CNES/Airbus Ds, USDA, AEX, Getmapping, Aerogrid, IGN, IGP, swisstopo, and the GIS User Community).*

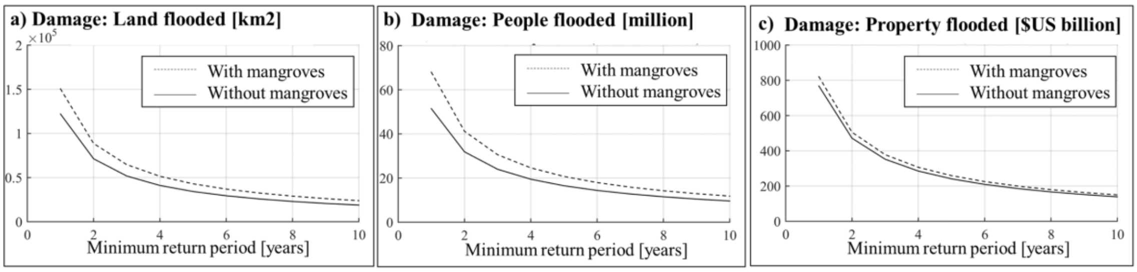

Supplementary Figure 15: *Expected annual flooded land, expected annual affected people and expected annual damage to stock under different initial limits of the return period (Tr_{min}) by using power law equation.*

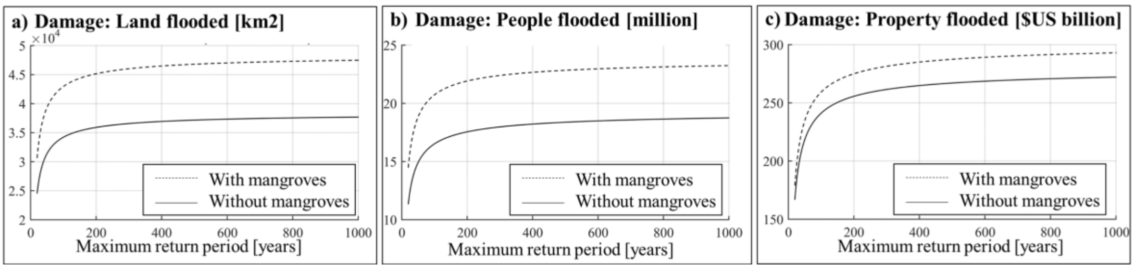

Supplementary Figure 16: Expected annual flooded land, expected annual affected people and expected annual damage to stock under different final limit of the return period (Tr_{max}) by using power law equation.

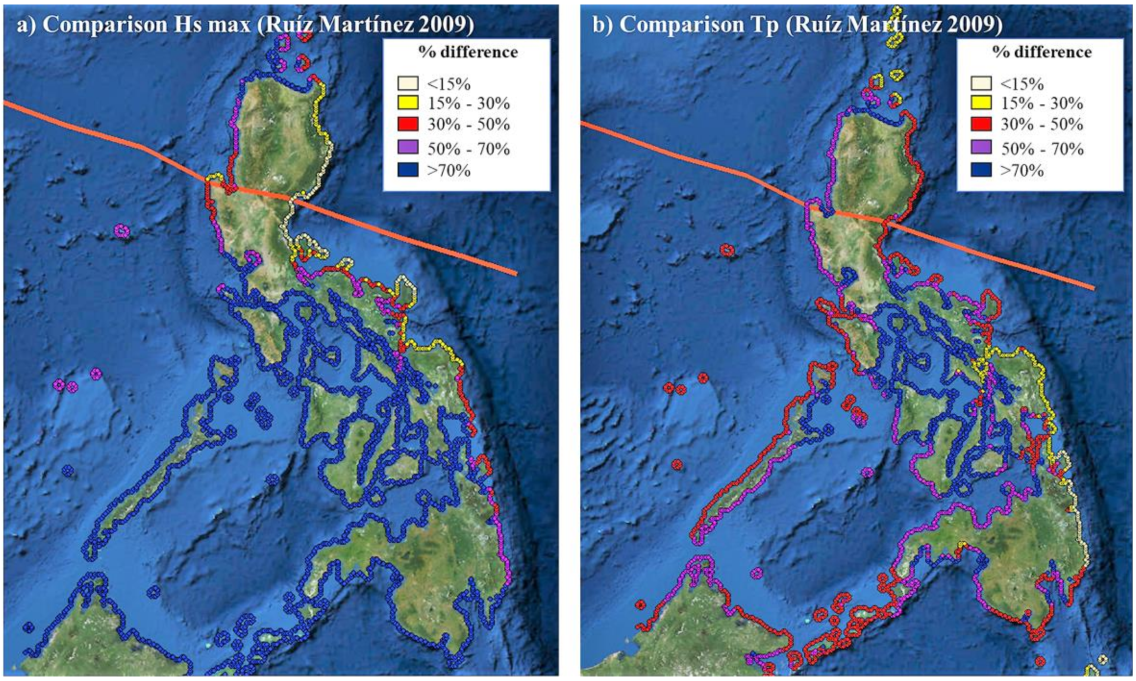

Supplementary Figure 17: Percentage difference between (a) the maximum wave height and (b) peak period calculated with our regression model of areas directly exposed to tropical cyclones (equations S.3 and S.4) versus Ruiz-Martinez (2009) model. *Figure created with ArcMap | ArcGIS Desktop software (10.7.1 version, http://desktop.arcgis.com/en/arcmap/). Online maps under a CC BY license, with permission from Esri, original Copyright 2018 Esri (Basemaps supported by Esri, DigitalGlobe, GeoEye, Earthstar Geographics, CNES/Airbus Ds, USDA, AEX, Getmapping, Aerogrid, IGN, IGP, swisstopo, and the GIS User Community).*

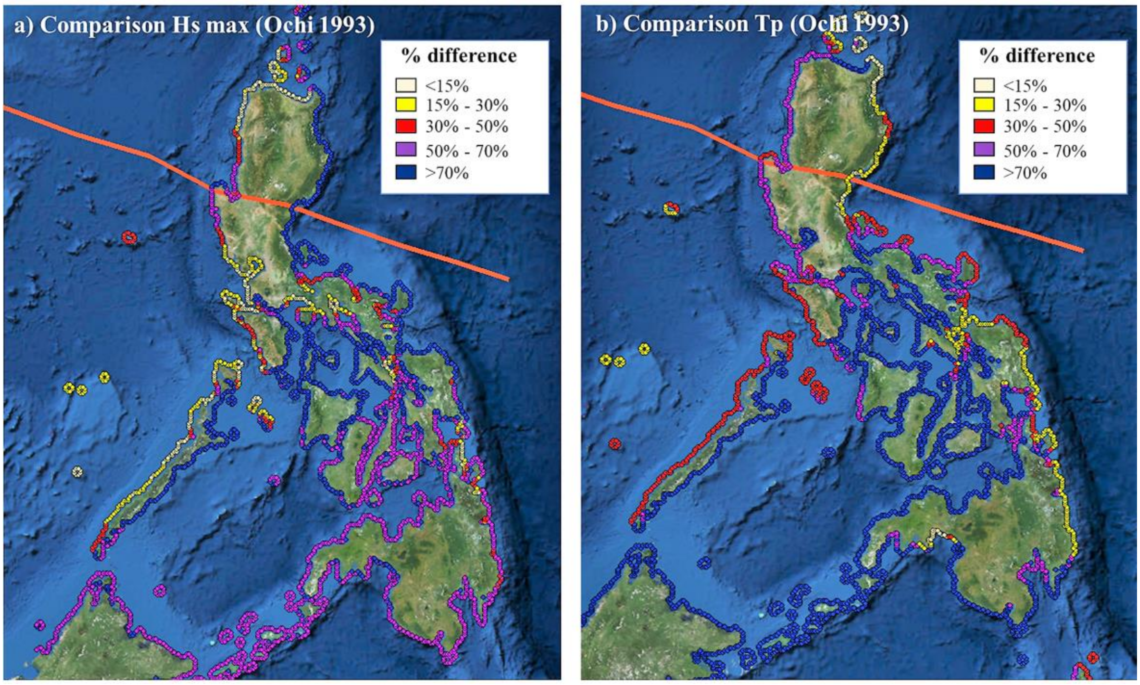

Supplementary Figure 18: Percentage difference between (a) the maximum wave height and (b) peak period calculated with our regression model of areas protected from tropical cyclones (equations S.7 and S.8) versus Ochi (2003) model. *Figure created with ArcMap | ArcGIS Desktop software (10.7.1 version, http://desktop.arcgis.com/en/arcmap/). Online maps under a CC BY license, with permission from Esri, original Copyright 2018 Esri (Basemaps supported by Esri, DigitalGlobe, GeoEye, Earthstar Geographics, CNES/Airbus Ds, USDA, AEX, Getmapping, Aerogrid, IGN, IGP, swisstopo, and the GIS User Community).*

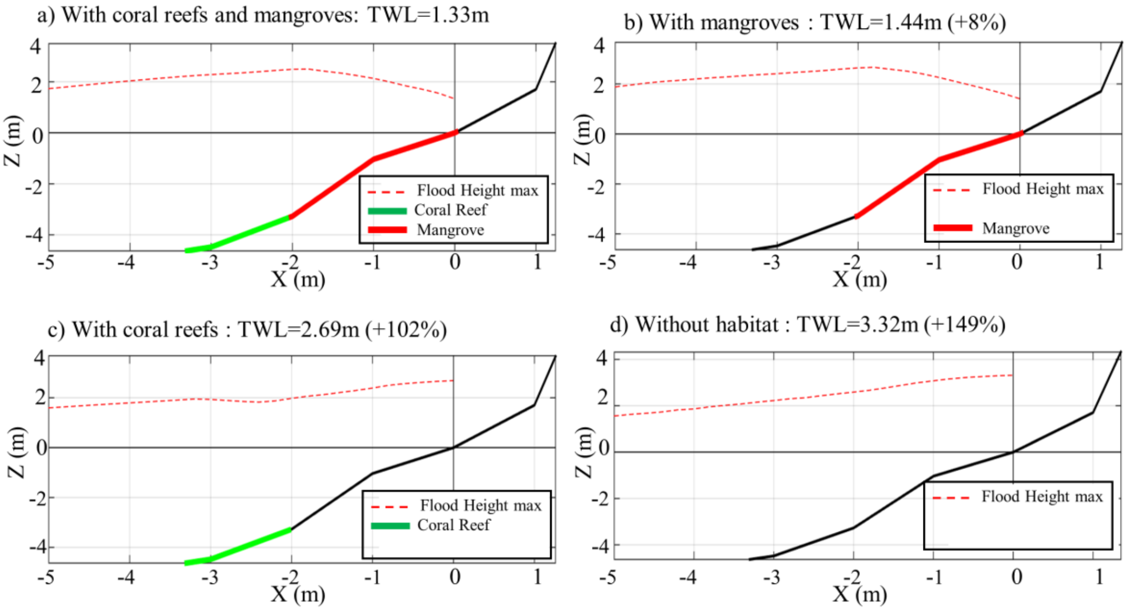

Supplementary Figure 19: Sensitivity analysis of Delft3D model under different presences of habitat. *The maximum flood height within an hour-long sea state defined by Hs=4.5m, Tp=20s and Storm Surge=1m is compared: a) in case of having both, coral reefs and mangroves, b) only mangroves, c) only coral reefs and d) with complete loss of the habitat.*

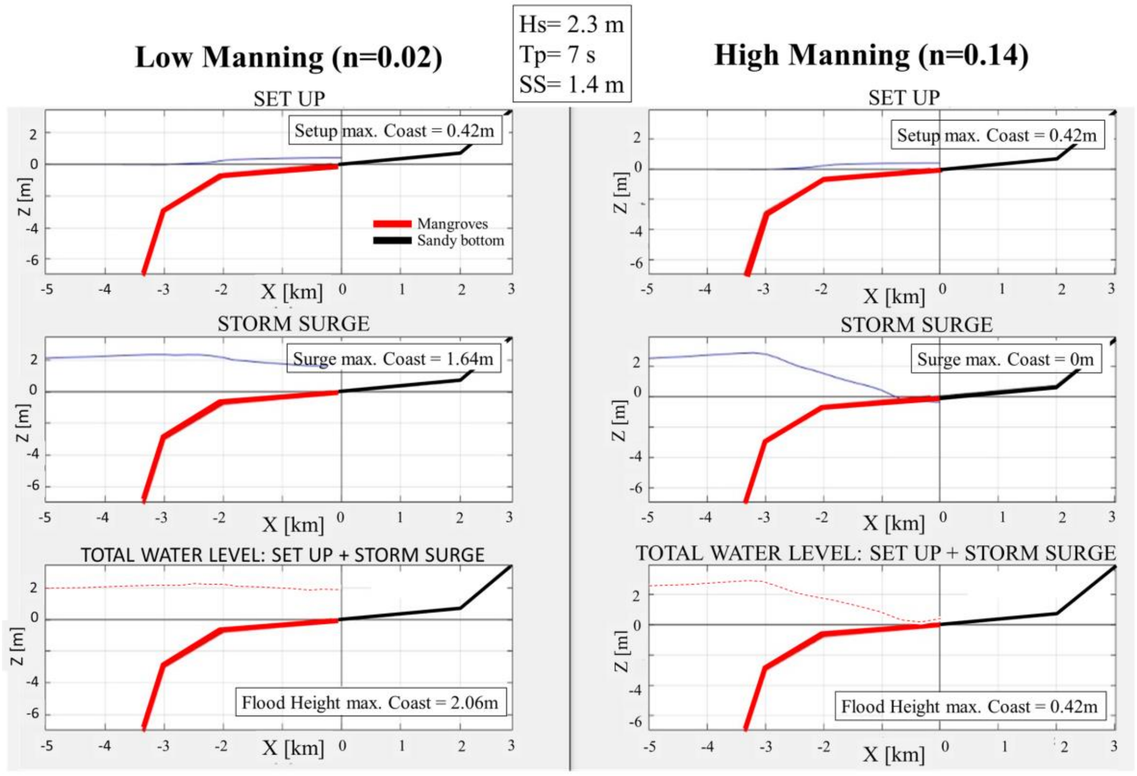

Supplementary Figure 20: Sensitivity analysis of the set-up, storm surge and Total Water Level in coast (i.e. flood height). *Two different values of the Manning coefficient (n) were tested: n=0.02 (left graphs) and n=0.14 (right graphs). The cross-shore propagations correspond to one single sea state defined by the offshore conditions of Hs=2.3m, Tp=7s and SS=1.4m.*

Supplementary Tables

Supplementary Table 1: T*ropical cyclones* e*xpected annual and return period flood damage (with and without mangroves) and benefit (without-with). Results are expressed in terms of land, people and property.*

	Land Flooded (x1000 km^2)			People Exposed (million)			Property Loss ($US billion)		
	With	Without	Benefit	With	Without	Benefit	With	Without	Benefit
Annual Expected	100	131	31	40	54	14	541	601	60
Tr=10	120	158	38	51	69	18	700	782	82
Tr=25	126	165	39	58	77	19	805	887	82
Tr=50	129	175	46	77	85	8	1108	1152	44
Tr=100	188	233	45	125	138	13	1785	1938	153

Supplementary Table 2: *Regular climate* e*xpected annual and return period flood damage (with and without mangroves) and benefit (without-with). Results are expressed in terms of land, people and property.*

	Land Flooded (x1000 km^2)			People Exposed (million)			Property Loss ($US billion)		
	With	Without	Benefit	With	Without	Benefit	With	Without	Benefit
Annual Expected	22	26	4	13	14	1	191	196	5
Tr=10	55	63	8	31	34	3	501	512	11
Tr=25	84	97	13	49	52	3	753	776	23
Tr=50	120	143	23	60	81	21	846	939	93
Tr=100	138	191	53	68	91	23	930	1046	116

Supplementary Table 3: *Top 15 ranked countries receiving the greatest yearly benefits from mangroves against tropical cyclones in terms of land, people, property and percentage of GDP lost.*

(a) Land [x1,000 km^2]			(b) People [million]			(c) Property [$US billion]			(d) Property/GDP [%]		
1	Cuba	3.70	1	Vietnam	4.46	1	United States	10.48	1	Belize	26.58
2	Bahamas	2.31	2	India	2.37	2	Taiwan	7.88	2	Suriname	21.35
3	Vietnam	1.89	3	Bangladesh	1.04	3	Mexico	7.24	3	Bahamas	12.79
4	Cambodia	1.59	4	Philippines	0.36	4	India	6.20	4	Mozambique	12.20
5	India	1.44	5	Brazil	0.33	5	Vietnam	3.54	5	Guyana	4.57
6	Nicaragua	1.40	6	Nigeria	0.30	6	China	2.77	6	Anguilla	4.55
7	United States	1.23	7	China	0.26	7	Saudi Arabia	1.61	7	Madagascar	3.43
8	Honduras	1.06	8	Mexico	0.22	8	Bahamas	1.44	8	Guinea Bissau	2.93
9	Mexico	0.99	9	Mozambique	0.22	9	Mozambique	1.34	9	Turks and Caicos	2.57
10	Bangladesh	0.77	10	Indonesia	0.21	10	Bangladesh	1.31	10	Sierra Leone	1.99
11	Indonesia	0.76	11	Ivory Coast	0.21	11	Brazil	0.72	11	Vietnam	1.72
12	Guyana	0.75	12	Ecuador	0.18	12	Suriname	0.70	12	Taiwan	1.71
13	Madagascar	0.67	13	Taiwan	0.17	13	Philippines	0.58	13	New Caledonia	0.96
14	Belize	0.66	14	Thailand	0.17	14	Cuba	0.58	14	Guinea	0.69
15	Mozambique	0.58	15	Pakistan	0.14	15	Australia	0.57	15	Mexico	0.69

Supplementary Table 4: *Top 15 ranked countries receiving the greatest yearly benefits from mangroves against regular climate in terms of land, people, property and percentage of GDP lost.*

(a) Land [x1,000 km^2]			(b) People [million]			(c) Property [$US billion]			(d) Property/GDP [%]		
1	Vietnam	1.22	1	Vietnam	2.562	1	China	0.58	1	Mozambique	5.40
2	Brazil	0.76	2	India	0.495	2	Vietnam	0.29	2	Belize	2.28
3	Ecuador	0.39	3	China	0.260	3	India	0.16	3	Vietnam	1.42
4	Cuba	0.22	4	Philippines	0.250	4	United States	0.83	4	Bahamas	0.94
5	Cambodia	0.19	5	Bangladesh	0.070	5	Mozambique	0.59	5	Palau	0.65
6	United States	0.19	6	Indonesia	0.029	6	Puerto Rico	0.59	6	Puerto Rico	0.56
7	India	0.19	7	Mozambique	0.023	7	Philippines	0.42	7	Solomon Islands	0.47
8	Bahamas	0.16	8	Australia	0.016	8	UAE	0.37	8	Antigua & Barbuda	0.39
9	Mexico	0.14	9	Thailand	0.011	9	Bangladesh	0.25	9	Vanuatu	0.34
10	Philippines	0.10	10	Puerto Rico	0.010	10	Australia	0.22	10	Guinea Bissau	0.32
11	Mozambique	0.10	11	Burma	0.009	11	Mexico	0.17	11	Micronesia	0.27
12	Colombia	0.10	12	Cambodia	0.009	12	Bahamas	0.10	12	New Caledonia	0.20
13	Indonesia	0.07	13	United States	0.006	13	Indonesia	0.56	13	Guinea	0.19
14	Guinea Bissau	0.06	14	Mexico	0.003	14	Burma	0.47	14	Liberia	0.19
15	Peru	0.06	15	Guinea	0.002	15	Belize	0.40	15	Madagascar	0.14

Supplementary Table 5: *Global average Flood Depth-Damage Functions for people and each different stock (residential, industrial). It represents the percentage of the exposed population and stock that is really damaged by flooding.*

Depth (m)	% Damage		
	Population	Residential stock	Industrial stock
0	0%	0%	0%
0.5	100%	33%	28%
1	100%	49%	48%

1.5	100%	62%	63%
2	100%	72%	72%
3	100%	87%	86%
4	100%	93%	91%
5	100%	98%	96%
6	100%	100%	100%

Supplementary Table 6: *Best predictor variables of ocean climate produced by tropical cyclones.*

(a) Coastal areas directly exposed to tropical cyclones	(b) Coastal areas protected from tropical cyclones
Hs_{max}= f (D_{min}, $\theta_{WP\_dist\_min}$, θ_{WP_mean}, V_{mean})<br>T_p = f (D_{min}, W_{dist_min}, θ_{WN_mean}, V_{wind_max})<br>SS_{max} = f (D_{min}, W_{dist_mean}, θ_{WP_media}, $\theta_{WN\_dist\_min}$)<br>T_{SSmax} = f (W_{mean}, $\theta_{WN\_dist\_min}$, D_{min}, V_{mean})	Hs_{max} = f (D_{min}, W_{dist_min}, V_{wind_max}, $\theta_{WN\_wind\_max}$)<br>T_p = f (W_{dist_min}, $\theta_{WN\_dist\_min}$, D_{min}, V_{mean})<br>SS_{max} = f (D_{min}, W_{dist_min}, V_{dist_min}, $\theta_{WN\_dist\_min}$)<br>T_{SSmax} = f (W_{mean}, D_{min}, V_{dist_min}, $\theta_{WN\_dist\_min}$)

Supplementary Table 7: *Best fit coefficient of power and logarithmic laws*

		Power: Y=aX^b			Log: Y=c logX + d		
		a	b	r^2	c	d	r^2
**Land flooded [km^2]**	With mangroves	$9.2206\cdot10^4$	0.2652	0.97	$6.3855\cdot10^4$	$1.6091\cdot10^4$	0.93
	Without mangroves	$1.1190\cdot10^5$	0.2782	0.97	$8.5545\cdot10^4$	$5.8094\cdot10^3$	0.92
	Benefits (without-with)	-	-	0.95	-	-	0.90
**People [mill.]**	With mangroves	$3.4254\cdot10^7$	0.3652	0.99	$4.6586\cdot10^7$	$-3.3880\cdot10^7$	0.94
	Without mangroves	$5.0557\cdot10^7$	0.3105	0.97	$4.8615\cdot10^7$	$-1.4653\cdot10^7$	0.94
	Benefits (without-with)	-	-	0.93	-	-	0.83
**Stock [$US bill.]**	With mangroves	$5.2307\cdot10^5$	0.3483	0.98	$6.3858\cdot10^5$	$-3.8380\cdot10^5$	0.94
	Without mangroves	$5.6366\cdot10^5$	0.3489	0.97	$6.9443\cdot10^5$	$-4.3019\cdot10^5$	0.93
	Benefits (without-with)	-	-	0.83	-	-	0.74

Supplementary Table 8: *Countries with less than 100 ha of mangroves excluded from the analysis*

**Country excluded**	**Ha of mangroves**	**Benefit/ha [$US]**
Benin	81	2 mill
Qatar	74	27,000
Martinique	63	0
Seychelles	63	16,000
British Virgin Islands	59	84,000
Samoa	54	0
Mauritius	40	0
St. Kitts and Nevis	31	96,000
Comoros	23	0
St. Vincent and Grenada	17	0
Saint Martin	11	0
Saint Barthelemy	9	0
Bahrain	5	68 mill
Aruba	3	0
Singapore	2	0

Supplementary Table 9: *Assumptions at each step of the methodology.*

		Assumptions and simplifications	Justification	References
**1**	**Offshore hydrodynamics**	• Split up high-intensity and low-intensity events	• Global reanalysis of waves and surge do not properly represent tropical cyclones.<br>• Tropical cyclones are local extreme events that must be modeled separately.	34,48
		• For low-intensity: Global reanalysis of waves and Storm Surge	• High quality offshore data is available globally (0.25º resolution for waves and 2º resolution for surge).<br>• Most of these data at the global scale are the same commonly used at local scales.	51,52
		• For high-intensity: Tropical cyclone modeling in the Philippines as baseline case	• The Philippines is the country that better represents any existing storm condition.<br>• Using one country as baseline case allows to run high resolution models (Delft3D).	34
		• Delft3D model for modeling offshore waves and surge in the Philippines	• Model validated with instrumental data (Supplementary Figure 10).	34
		• Predictive model to estimate Hs, Tp and SS offshore worldwide	• Sensitivity analysis of the predictive model using different predictive variables. Best predicted variables chosen based on Pearson coefficient (Supplementary Table 6).<br>• Different regression model for coastal areas protected from tropical cyclones and non-protected.<br>• No overfitting assured: model built with 90% of the Philippine´s data (another 10% reserved for validation).<br>• Model validated with existing empirical formulations (e.g. Ochi 1993, Ruiz 2009, Supplementary Figure 16 and 17).	71
**2**	**Nearshore hydrodynamics**	• Propagation with Snell's law	• Accounts for waves transformation: height and directionality.<br>• Low computational cost.	32
		• Selection method to reduce the number of sea states of low-intensity events	• Reduce computational cost.<br>• Validated and applied since 2011 (Camus et al., 2011a, 2011b).	50,51,63,64,72
**3**	**Mangrove effect on waves and surge propagation**	• Interpolation tables from the Philippines to estimate flood height	• The Philippines is the country that better represents any existing storm condition and mangrove characteristics (see Supplementary Figures 7 and 8).<br>• Using one country as baseline case allows to run high resolution models (Delft3D).	29
		• Mangrove model 1D (cross-shore profiles)	• Low computational cost.<br>• High resolution cross-shore profiles (every 2 km of coastline) oriented parallel to the nearshore bathymetry to reduce the errors from not considering 2D effects of wave propagation (e.g. diffraction).	29,32

			• High resolution process-based numerical model 1D simulations (Delft3D) accounting for breaking and friction.	
		• Global Mangrove roughness modeling based on Manning coefficient	• Simple representation of ecosystems, only by its roughness.<br>• Sensitivity analysis done, testing the effect of Manning coefficient in the flood height (Supplementary Figure 19).	[34,58]
		• RBF method: Reconstructions of historical time series of flood height over the full period	• Full time series reconstruction required for stochastic risk analysis.<br>• Widely validated and used RBF method for time series reconstruction.	[34,65]
**4**	**Estimate Flooding**	• Modified bathtub approach with hydraulic connectivity	• Low computational cost.<br>• Large scale domains (i.e. global analysis)..<br>• Similar results than using high resolution process-based flood models if using global resolution topography (i.e. 90m)<br>• Uncoupled calculation of flood height and flooding inland provides future flexibility in re-running only flooding models (e.g., if new high-resolution topography available).	[32,34]
		• Topography: Global SRTM ~90m	• Global consistency in the analysis (same resolution and same data source worldwide).<br>• Globally accepted database. Applied in several flood risk analysis.	[32,34,73]
		• Stationary Extreme value analysis	• Maximum envelopes of coastal flooding.	[32,34]
**5**	**Exposure, Damage and Risk**	• Global data of population and stock	• Global consistency in the analysis (same resolution and same data source worldwide).<br>• Globally accepted database and widely applied in risk flood assessments.	[69]
		• Global damage functions	• Global consistency in the analysis.<br>• Consider damage to people (nº of people really affected by flooding).	[3,49,74]
		• Annual Expected Damage functions truncated between Tr=5yr and Tr=100yr	• Sensitivity analysis of the Annual Expected Damages obtained using different upper and lower limits (Supplementary Figure 15).<br>• Sensitivity analysis of fitting the return period curve to potential law equation vs logarithmic law equation (Supplementary Table 6).	[32,34]
